# Atomistic View of the Energy Transfer in a Fluorophore-Functionalized Gold Nanocluster

**DOI:** 10.1021/jacs.3c02292

**Published:** 2023-06-28

**Authors:** Kyunglim Pyo, María Francisca Matus, Eero Hulkko, Pasi Myllyperkiö, Sami Malola, Tatu Kumpulainen, Hannu Häkkinen, Mika Pettersson

**Affiliations:** † Nanoscience Center, Department of Chemistry, P.O. Box 35, University of Jyväskylä, Jyväskylä FI-40014, Finland.; b Nanoscience Center, Department of Physics, P.O. Box 35, University of Jyväskylä, Jyväskylä FI-40014, Finland.; c Nanoscience Center, Department of Biological and Environmental Sciences, P.O. Box 35, FI-40014, University of Jyväskylä, Jyväskylä FI-40014, Finland

## Abstract

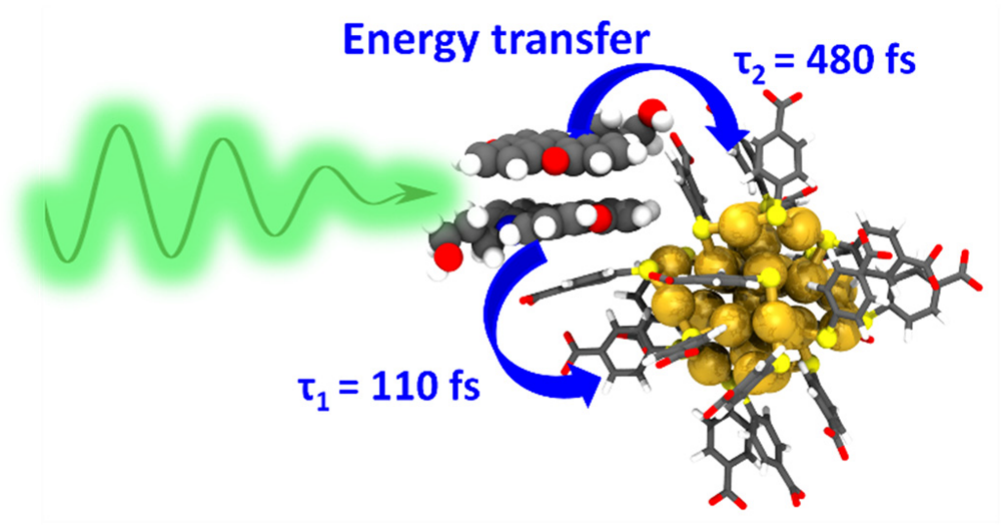

Understanding the dynamics of Förster resonance energy transfer (FRET) in fluorophore-functionalized nanomaterials is critical for developing and utilizing such materials in biomedical imaging and optical sensing applications. However, structural dynamics of noncovalently bound systems have a significant effect on the FRET properties affecting their applications in solutions. Here, we study the dynamics of the FRET in atomistic detail by disclosing the structural dynamics of the noncovalently bound azadioxotriangulenium dye (KU) and atomically precise gold nanocluster (Au_25_(*p*-MBA)_18_, *p*-MBA = *para*-mercaptobenzoic acid) with a combination of experimental and computational methods. Two distinct subpopulations involved in the energy transfer process between the KU dye and the Au_25_(*p*-MBA)_18_ nanoclusters were resolved by time-resolved fluorescence experiments. Molecular dynamics simulations revealed that KU is bound to the surface of Au_25_(*p*-MBA)_18_ by interacting with the *p*-MBA ligands as a monomer and as a π–π stacked dimer where the center-to-center distance of the monomers to Au_25_(*p*-MBA)_18_ is separated by ∼0.2 nm, thus explaining the experimental observations. The ratio of the observed energy transfer rates was in reasonably good agreement with the well-known 1/*R*^6^ distance dependence for FRET. This work discloses the structural dynamics of the noncovalently bound nanocluster-based system in water solution, providing new insight into the dynamics and energy transfer mechanism of the fluorophore-functionalized gold nanocluster at an atomistic level.

## Introduction

Fluorophore-functionalized nanomaterials utilizing the Förster resonance energy transfer (FRET) process have received considerable research interest in the past decade. In several recent studies, thiolate ligand-protected metal nanoclusters were selected as the fluorophore-functionalized nanomaterials due to their low toxicity, facile surface functionalization, high photostability, and well-defined structure.^1−7^ Moreover, their broad absorption range has made metal nanoclusters ideal materials for FRET-based systems. In these systems, the fluorescence efficiency of the metal nanocluster is enhanced by the energy transfer from the fluorophore, which enables utilization of the nanocluster in many optical applications, such as bioimaging and light-emitting diodes (LED).^8−10^ Furthermore, the size and the surface charge of the nanoclusters modulate the fluorescence in pH sensing applications by adjusting the pH response range of the fluorophore.^11,12^

Despite these merits, a detailed understanding of the dynamics of the nanocluster–fluorophore energy transfer pair in solution is lacking. For example, it is known that different counterions, concentrations, and temperatures can change the behavior of small ligand-protected metal nanoclusters and fluorophores, such as the orientation and rigidity of the ligands or the degree of aggregation.^13−18^ These structural changes can alter the solubility, absorbance, and emission properties of the material, which will affect the fluorescence lifetime of the donor. As a result, the energy transfer properties are likely to be influenced, compromising the utilization of these materials in applications. In small systems based on nanoclusters or fluorophores, minor changes can dramatically affect the properties. Therefore, it is necessary to investigate the structural dynamics and interaction behavior between the fluorophores and nanoclusters. By using FRET analysis, there have been attempts to study the structural dynamics of biomolecules, such as protein conformation change, DNA cleavage, and molecular level interactions.^19−23^ However, to the best of our knowledge, there still has not been any in-depth analysis of the structural dynamics between metal nanoclusters and fluorophores in solutions. Only studies on enhancing the energy transfer efficiency for further applications or analyzing the excited-state dynamics by measuring the transient absorption spectroscopy have been reported.^9,24,25^

In this work, we present a detailed, atomistic scale study of structural dynamics of a noncovalently bound gold nanocluster (Au_25_(*p*-MBA)_18_) and fluorophore (azadioxotriangulenium dye, KU) by combining steady-state and time-resolved spectroscopies, Molecular dynamics (MD) simulations, and NMR spectroscopy. First, the yield of energy transfer was determined from steady-state luminescence measurements. Second, broadband fluorescence up-conversion spectroscopy (FLUPS) with femtosecond time resolution enabled us to measure the full time-dependent fluorescence spectrum of the KU dye. Surprisingly, two femtosecond lifetime components were observed for the bound KU dye, which have not been reported before. Further investigation using MD simulations showed that there are two KU dye configurations interacting with the deprotonated *p*-MBA ligands of the Au_25_(*p*-MBA)_18_ in solution: a monomer and a dimer. The observed distance between the KU dyes in its dimer configuration could explain the two observed lifetime components, also supported by the Förster theory calculation. Concentration-dependent FLUPS measurements gave additional support for the dimer hypothesis. These results evidently confirm that the fluorescence lifetimes of the dyes are strongly associated with the intermolecular interaction, and therefore, it is inevitable to study the configuration of the total system. This research gives a detailed picture of the dynamics of the energy transfer mechanism for a nanocluster–fluorophore system and reveals a surprising dimer mechanism of energy transfer.

## Results and Discussion

### Steady-State Measurements

**Figure 1:**
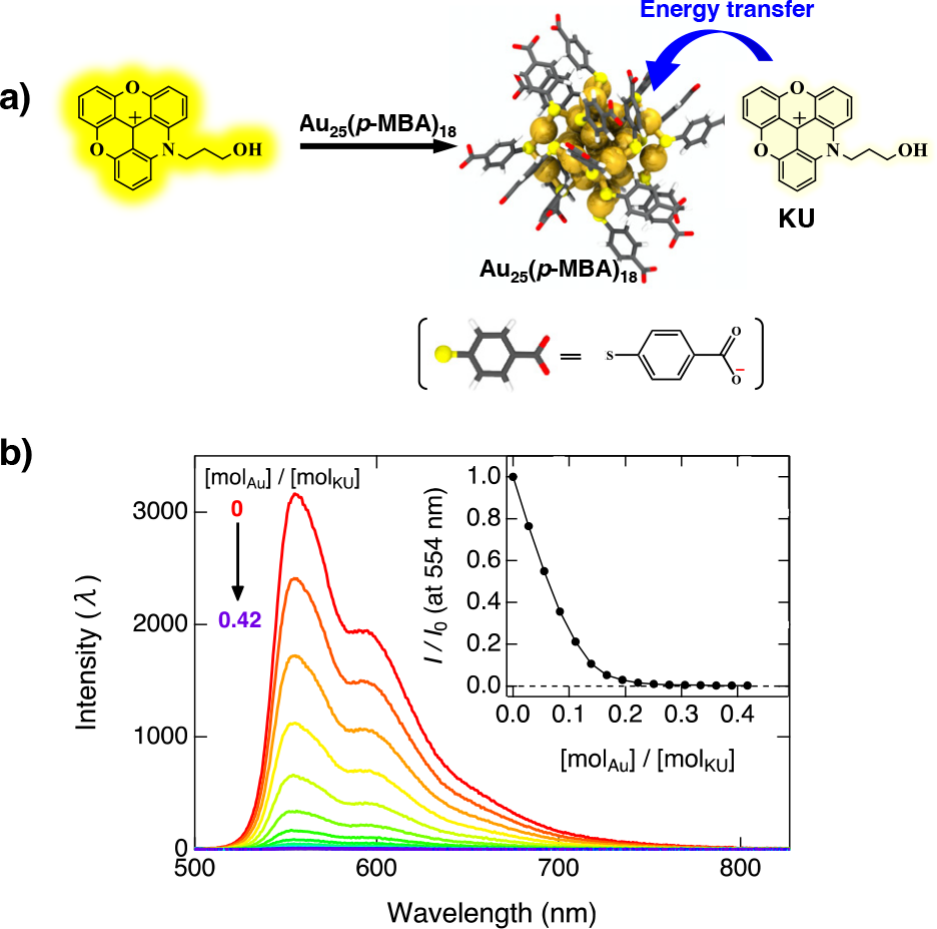
(a) Schematic diagram illustrating the interaction and energy transfer process between the Au_25_(*p*-MBA)_18_ nanocluster and KU dye in basic solution. (b) Fluorescence spectra of KU in different molar ratios of Au_25_(*p*-MBA)_18_ at pH 10. The inset shows the relative intensity at 554 nm as a function of [mol_Au_]/[mol_KU_] molar ratio.

Au_25_(*p*-MBA)_18_ is known to possess a negative surface charge in basic conditions due to deprotonation of the carboxylic group of the *p*-MBA ligands, promoting the binding of the positively charged KU dyes to form a noncovalently bound complex (Au_25_(*p*-MBA)_18_+KU).^11,12^ (Figure 1a) The complexation can facilitate Förster-type energy transfer from the KU dye to the gold nanocluster, and as a result, the emission of the KU dye decreases rapidly upon the addition of Au_25_(*p*-MBA)_18_ in aqueous solution. Steady-state fluorescence spectra of a KU dye solution (pH 10; see Supporting Information for experimental details) upon addition of Au_25_(*p*-MBA)_18_ are presented in Figure 1b. In our previous study, the fluorescence quenching was explained by a FRET process from the excited KU dye to Au_25_(*p*-MBA)_18_,^11,12^ facilitated by the proximity and significant spectral overlap between the absorption and emission spectra of Au_25_(*p*-MBA)_18_ and the KU dye, respectively (Figure S2).

**Figure 2:**
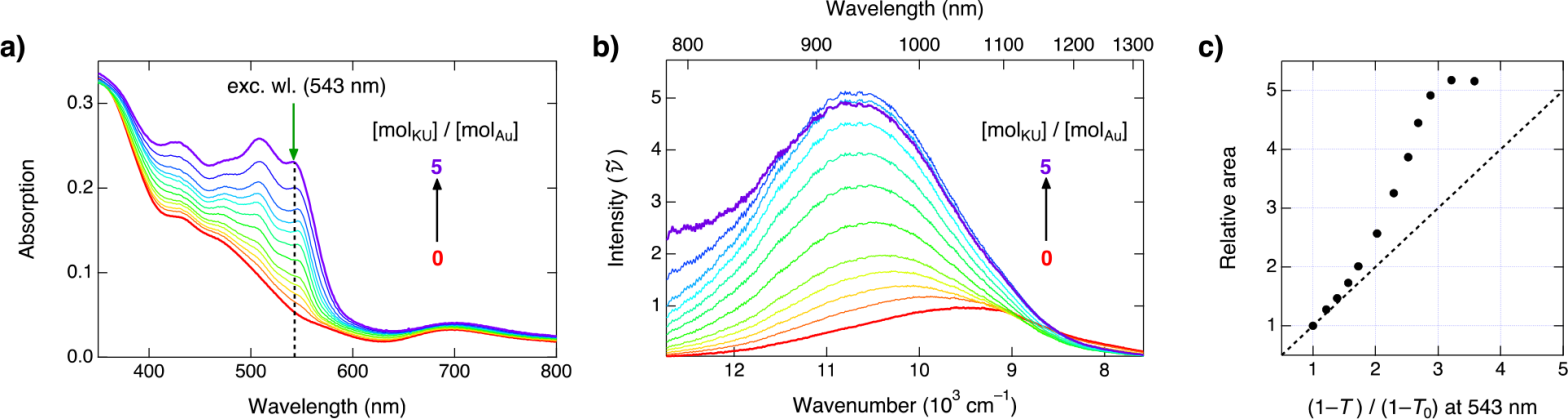
(a) Absorption spectra of Au_25_(*p*-MBA)_18_ (*c* = 5.8 μM) upon addition of KU. (b) Corresponding luminescence spectra in wavenumbers at the excitation wavelength of 543 nm indicated by the green arrow in (a). (c) Relative areas of the luminescence bands as a function of the relative absorbance at the excitation wavelength. The dashed line represents the linear correlation.

To gain further support for the energy transfer mechanism, we now measured the luminescence spectra of Au_25_(*p*-MBA)_18_ upon addition of KU dye in the NIR region using a home-built spectrometer (see Supporting Information for experimental details). The sample was excited at 543 nm, which corresponds to the low-energy absorption maximum of the KU dye. In the titration, the concentration of the Au_25_(*p*-MBA)_18_ was held constant (*c*_Au_ = 5.8 μM) while the amount of KU was increased up to ca. 28 μM, corresponding to about [mol_KU_]/[mol_Au25_] = 5. The absorption and luminescence spectra are presented in Figure 2a and 2b, respectively. The spectra were fitted with a Gaussian band-shape function in the wavenumber domain (Figure S4) to extract the luminescence intensities, and the relative Gaussian-band areas as a function of relative absorptance (*f* = 1 – *T* = 1 – 10^–*A*^) at the excitation wavelength are presented in Figure 2c.

The luminescence spectra of Au_25_(*p*-MBA)_18_ reveal several interesting changes upon the addition of the KU dye. First, a clear luminescence enhancement and a blueshift of about 1000 cm^–1^ are observed (Figure S4). Up to a molar ratio of about 1:1 ([mol_Au25_]/[mol_KU_]), the enhancement is nearly linear with a slope of unity, after which the enhancement becomes significantly stronger. The enhancement starts plateauing after exceeding a molar ratio of about 1:4 and eventually decreases above a molar ratio of 1:5 accompanied by an appearance of the fluorescence tail of the unquenched KU below 850 nm, in agreement with the titration presented in Figure 1.

The luminescence area is expected to increase linearly as a function of absorptance in dilute solutions, where inner filter effects can be largely ignored.^26,27^ Here, the enhancement is significantly stronger and cannot be explained solely by the energy transfer. It has been reported for different gold clusters that rigidifying the ligands results in a blueshift and enhancement of the luminescence.^17,24^ Association of the large aromatic KU dye on Au_25_(*p*-MBA)_18_ appears to induce a rigidifying effect similar to that particularly evident upon association of two or more dyes on a single Au_25_ cluster. From the current experiment, it is not possible to separate the contributions from energy transfer and the rigidifying effect on the overall enhancement.

**Figure 3:**
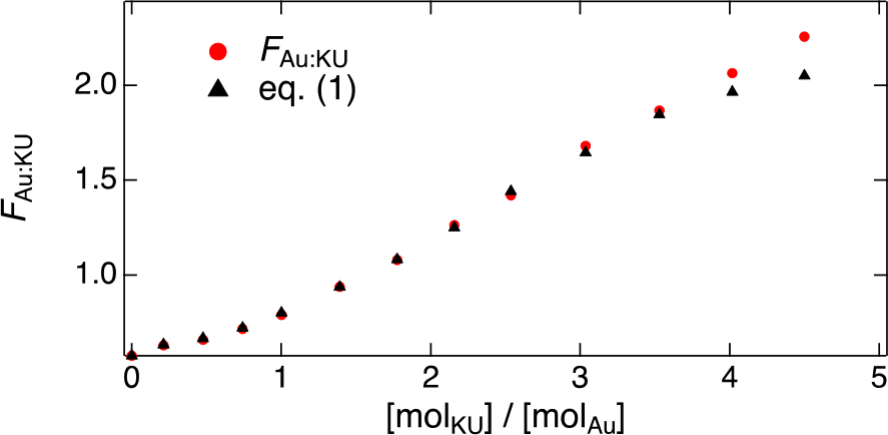
Observed (red circles) total luminescence yield of Au_25_(*p*-MBA)_18_ upon 543 nm excitation together with the yield calculated (black triangles) according to eq 1 with an energy transfer efficiency of 81%.

To overcome this problem, we performed a comparative experiment where we measured the luminescence enhancement of the Au_25_(*p*-MBA)_18_ upon addition of KU at two different excitation wavelengths (543 and 632 nm). The absorption spectrum of the KU dye does not extend to 632 nm, and therefore we could estimate the relative luminescence quantum yield (ϕ_Au_) of the Au_25_(*p*-MBA)_18_ at each KU concentration following the protocol presented in ref (26) (see Supporting Information for details). According to the data, the quantum yield increases by about 41% upon addition of 4 equiv of KU (Figure S6). The total luminescence of the complex at 543 nm excitation can be now represented in terms of the quantum yield considering both direct and indirect excitation of the gold cluster according to
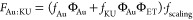
1where *f*_Au_ and *f*_KU_ are the total absorptance values scaled by the relative absorption probabilities of Au_25_(*p*-MBA)_18_ and KU at 543 nm, ϕ_Au_ is the relative luminescence quantum yield of Au_25_(*p*-MBA)_18_, ϕ_ET_ is the energy transfer efficiency, and *f*_scaling_ is an instrument-related scaling factor (see Supporting Information for details). The measured and calculated total luminescence yields as functions of [mol_KU_]/[mol_Au_] are presented in Figure 3.

Best agreement between the measured and estimated luminescence integrals was obtained with a ϕ_ET_ value of 81%. The agreement between the observed and calculated luminescence yields is excellent up to a molar of about 3.5, after which the fluorescence of free KU dye starts to contribute to the observed luminescence yield. The nearly quantitative quenching of the KU fluorescence below this molar ratio additionally suggests the presence of a competing quenching mechanism, most probably an electron transfer process.

The second interesting observation concerns the shape of the Au_25_(*p*-MBA)_18_ luminescence band. Our spectrum consists of a single band, which in the absence of KU is slightly asymmetric but upon addition of KU is well represented by a Gaussian band-shape function (Figure S4). The spectra are qualitatively different from a recently published spectrum of Au_25_(*p*-MBA)_18_ that displayed two distinct bands with a well-defined minimum at around 975 nm.^28^ Moreover, the authors observed that the relative intensities of the two bands changed upon changing the ligands or on association of the gold clusters with proteins. We wish to point out that the minima observed in all the reported spectra coincide with an absorption band of water. In a conventional spectrometer with right-angle geometry, the absorption of the solvent can give rise to such a minimum in the observed spectra. Furthermore, spectral shifts could be manifested as changes in relative intensities. The spectra presented here were collected in front-face geometry and are thus less sensitive to the absorption of the solvent. At the same time, quantifying the magnitude of the inner filter effect is more challenging and was not accounted for in our measurement.

The steady-state luminescence data provide limited information about the actual dynamics of the complex. The key experimental parameter is the fluorescence decay time, which can be related to the distance between the fluorophore and the cluster. Hulkko et al. made an attempt to measure the fluorescence decay time of a covalently bound Au_102_-KU hybrid cluster in different pH with picosecond time-correlated single photon counting.^11^ In acidic conditions, the covalently bound KU dye dissociated from the gold nanoclusters, forming a Au_102_+KU complex. Hulkko et al. found a 180 ps component attributed to the complexed KU and a 19.1 ns component attributed to an unbound KU. However, the fast decay component was instrument-limited, and thus, the true decay time and, therefore, the distance between the KU dye and the cluster remained unresolved.^11^ Determination of the true decay time of the KU dye is a key step for unraveling the structural dynamics of the complex in solution. Therefore, we carried out broadband FLUPS^29,30^ measurements to solve the true decay time of the KU dye in the presence of atomically precise Au_25_(*p*-MBA)_18_ nanoclusters in order to gain a more profound understanding of the dynamics in the Au_25_(*p*-MBA)_18_+KU complex system.

### FLUPS Measurement of the Au_25_(*p*-MBA)_18_+KU Complex

**Figure 4:**
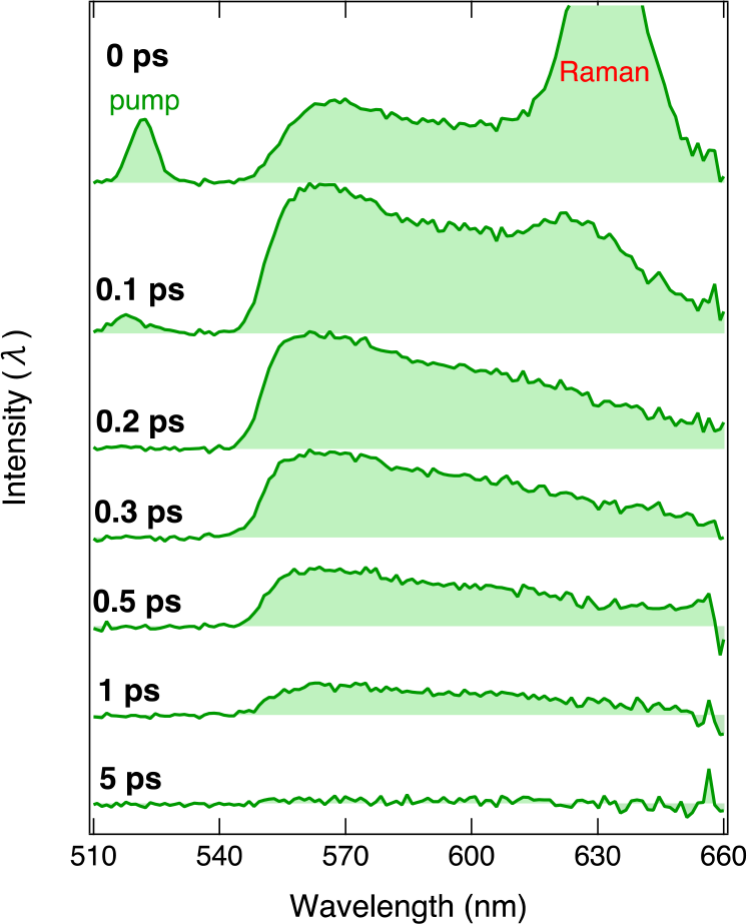
Time-resolved fluorescence spectra of the Au_25_(*p*-MBA)_18_+KU complex with a 1:2 molar ratio ([KU] = 0.2 mM) at pH 11. The spectra are measured with a 550 nm long-pass filter to suppress the scattered excitation light at 520 nm. The intense signal at 635 nm observed in the early spectra is due to Raman scattering of excitation light.

FLUPS measurements were carried out at a molar ratio of 2 ([mol_Au_]/[mol_KU_] = 1:2), at which the steady-state fluorescence of the KU dye is completely quenched (Figure 1b). Therefore, the concentration of the unbound KU dye is expected to be negligible. The fluorescence spectra were recorded as a function of time upon direct excitation of the low-energy absorption band of the KU dye at 520 nm (Figure S3). Absolute concentrations were adjusted to achieve a KU dye absorption of about 0.2 in a 1 mm cuvette at the excitation wavelength. The time-resolved fluorescence spectra of the Au_25_(*p*-MBA)_18_+KU complex with 1:2 molar ratio at pH 11 are presented in Figure 4. Full experimental details and data processing methods are given in the Supporting Information.

The time-resolved spectra show the appearance of a broad emission band with a maximum at 565 nm and a shoulder at 600 nm, in agreement with the steady-state fluorescence (Figure 1b). However, the 565 nm band is significantly distorted due to the presence of the 550 nm long-pass filter that was used to suppress the scattered excitation light. A reference KU sample in the absence of Au_25_(*p*-MBA)_18_ shows nearly identical spectra, demonstrating that the fluorescence signal originates solely from the KU dye (Figure S7). In addition to the KU fluorescence, residual excitation light and a strong Raman signal are visible in the early spectra at 520 and 635 nm, respectively. After the appearance, the overall fluorescence decays rapidly in a few picoseconds, in contrast to the slow excited-state decay observed for the reference KU sample in the absence of Au_25_(*p*-MBA)_18_ (Figure S8).

Time-resolved spectra of the complex did not exhibit significant spectral evolution (Figure S8). Therefore, the fluorescence decay was extracted from an integrated fluorescence signal between 560 and 610 nm and analyzed with a three-exponential function convolved with a Gaussian-simulated instrument response function (IRF). The analysis reveals two major decay components with decay times of 110 ± 20 and 480 ± 70 fs, in addition to a longer (3.5 ± 0.5 ps) minor component. Due to the rather weak signal and fast decay components, the experiments were repeated several times on slightly different experimental conditions (excitation power/wavelength, concentration, pH). All independently measured decays exhibit two major decay components with ca. 100 and 500 fs lifetimes. This strongly suggests that the two lifetimes can be associated with two distinct subpopulations in the complexes.

Interestingly, the reference KU sample also shows a slight (20%) fluorescence decay between 550 and 600 nm during the first 10 ps, whereas the intensity above 610 nm remains constant in the 20 ps time window (Figure S9). This suggests that the decay at the high-energy side results in emission on the low-energy side with a lower transition dipole moment. Such behavior is commonly observed for excimer formations in H-type aggregates, resulting in weak emission in the longer wavelength region and has been also reported for related triangulenium dyes.^31,32^ Moreover, evidence for dimer formation (or aggregation) between the ground-state species of the KU dye was also observed in NMR spectra measured in the same concentration range as the FLUPS measurements (Figure S10 and Table S1 and Supporting Information for details).

**Figure 5:**
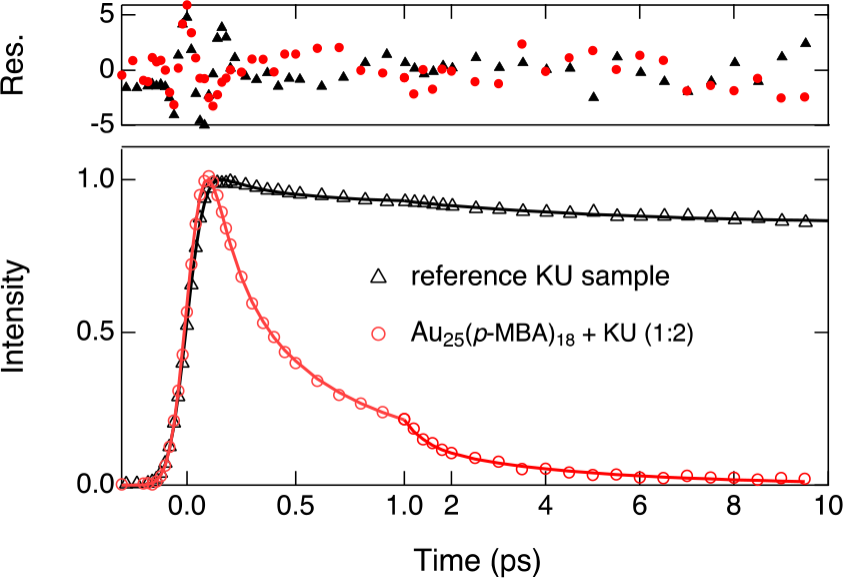
Fluorescence decays of the reference KU sample (black) and the Au_25_(*p*-MBA)_18_+KU complex with a 1:2 molar ratio (red). The solid lines represent fits with a three-exponential function. Residuals in standard deviations are given in the top panel. The decays were obtained by integrating the fluorescence signal in the ranges 560–590 and 560–610 nm for the reference KU sample and the complex, respectively.

Fluorescence decay constants of the reference KU sample were extracted from an integrated signal between 560 and 590 nm, yielding a biexponential decay (Figure 5) with time constants and relative amplitudes of 230 ± 70 fs (11%) and 4.8 ± 1.1 ps (9%) followed by the long-lived fluorescence of the unquenched KU dye. The fast decay component, tentatively attributed to excimer formation, is on the same time scale as the short decay components observed for the Au_25_(*p*-MBA)_18_+KU complex. However, the lack of any appreciable spectral changes in the fluorescence of the Au_25_(*p*-MBA)_18_+KU complex as well as rapid decay of the total fluorescence intensity suggests that the nature of the quenching mechanism is different and cannot be attributed to excimer formation. Therefore, energy transfer from the excited KU dye to the gold nanocluster is suggested to be the dominant quenching pathway in the Au_25_(*p*-MBA)_18_+KU complex, which is also supported by the steady-state luminescence measurements.

### Binding Modes of the Au_25_(*p*-MBA)_18_+KU Complex

Atomistic simulation methods have provided valuable guidance in understanding different physicochemical properties of ligand-protected metal nanoclusters, such as their geometry, stability, surface charge, and solubility, and how they behave under specific environmental conditions.^12,33−36^ Here, we explored the potential interaction modes between the KU dyes and Au_25_(*p*-MBA)_18_ nanocluster by modeling a dynamic formation of the Au_25_(*p*-MBA)_18_+KU complex in aqueous solution using GROMACS software.^37^ We considered three different starting configurations for complex formation with a 1:2 ([mol_Au25_]/[mol_KU_]) molar ratio (Figure S11) to represent the random positions of the dye molecules when approaching the Au_25_(*p*-MBA)_18_ nanocluster.

**Figure 6:**
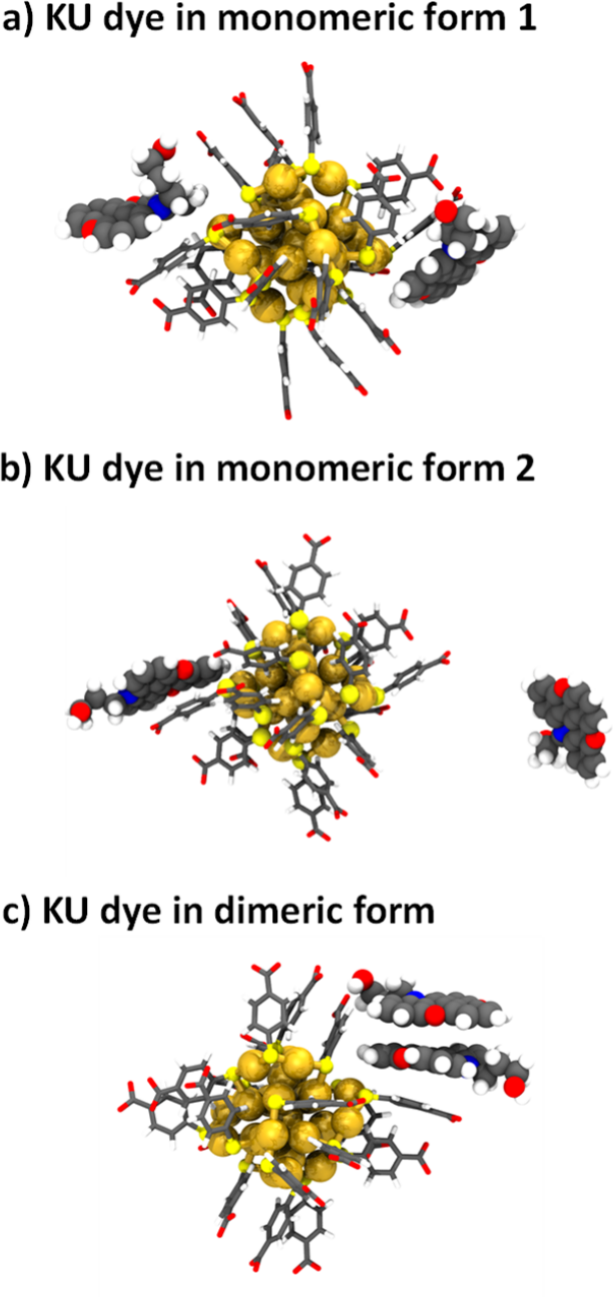
Representative snapshots from 500 ns molecular dynamics simulations showing the binding modes of KU dyes on the Au_25_(*p*-MBA)_18_ surface as (a) monomers when both dyes remain equidistant from the nanocluster, (b) monomers when only one KU dye interacts with the ligand layer of the nanocluster, or (c) stacked dimer.

Figure 6 shows the main interaction modes between the KU dye and the ligand layer of Au_25_(*p*-MBA)_18_ observed in 500 ns MD trajectories. The Au_25_(*p*-MBA)_18_+KU complex is established when the KU dyes interact with the deprotonated *p*-MBA ligands either as monomers (Figure 6a and b) or after dimerization (Figure 6c). KU dimer formation is mediated by aromatic stacking, while the interaction between KU dyes (in either their monomeric or dimeric form) and *p*-MBA ligands is influenced by a combination of hydrogen bonding and aromatic π-stacking (Figures S12–S14).

Generally, the interactions between the dyes and the nanocluster are dynamic, and once formed, the dye–cluster complex can break and reform. However, several extended periods were observed in which two KU dyes were independently bound to the nanocluster at different sites. For the stacked KU dyes, the interaction with the cluster seemed to be more stable, and once formed the dye–cluster complex remained stable to the end of the run (Figure S14). We also started simulations from an initial condition where the KU dyes had a T-stacking interaction with three stacked *p*-MBA ligands (see Supporting Information for details) but found that T-stacking was not stable during the simulation.

We note that the calculated distances between the Au_25_(*p*-MBA)_18_ nanocluster (central atom from the Au_25_(*p*-MBA)_18_ core) and the KU dyes (central carbon from the aromatic portion) are virtually identical in cases where the two dyes are independently bound as monomers. However, in the case of the stacked dimer, the distances have well-defined, distinct values of 1.1 ± 0.04 and 1.3 ± 0.07 nm averaged over the MD trajectories, thus having an average difference of ∼0.2 nm. Due to strong dependency of the energy transfer rate on the separation distance, the two individual KU dyes of the stacked dimer can explain the two measured, distinct fluorescence decay times. These findings suggest that the two stacked KU dyes interact independently with the nanocluster.

### FLUPS Measurement with Different Au_25_(*p*-MBA)_18_:KU Ratios

**Figure 7:**
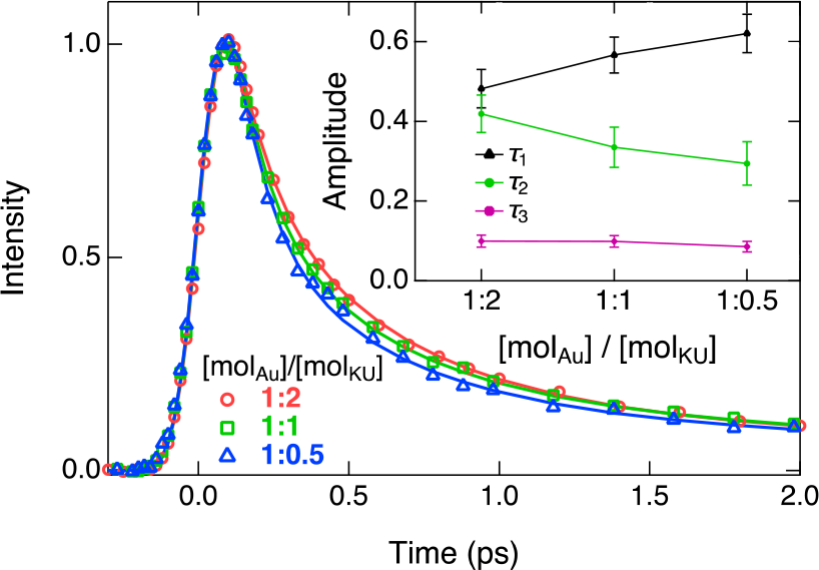
Fluorescence decays of the Au_25_(*p*-MBA)_18_+KU complex with 1:2, 1:1, and 1:0.5 molar ratios with a constant concentration of Au_25_(*p*-MBA)_18_ (0.1 mM) at pH 11. The solid lines represent global fits with a three-exponential function with lifetimes of τ_1_ = 110 ± 20 fs, τ_2_ = 480 ± 70 fs, and τ_3_ = 3.5 ± 0.5 ps. The relative amplitudes of the decay components are given in the inset.

Based on the MD simulations, the two lifetimes observed in the FLUPS measurements were rationalized by competitive binding of KU monomers and dimers on the Au_25_(*p*-MBA)_18_ clusters. Since the monomers and dimers are in dynamic equilibrium in solutions, the relative amounts of the bound monomer and dimer are expected to be dependent on the absolute concentration of the KU dye. Therefore, we performed comparative FLUPS experiments where the absolute concentration of KU was varied while maintaining a constant Au_25_(*p*-MBA)_18_ concentration of 0.1 mM. Molar ratios ([mol_Au25_]/[mol_KU_]) of the samples were 1:1 and 1:0.5, corresponding to 2-fold and 4-fold lower concentrations of the KU dye compared to the 1:2 molar ratio. In order to extract the relative amplitudes reliably, all three samples were measured with identical experimental conditions. Time-resolved spectra are given in the Supporting Information (Figure S8). The fluorescence signals were integrated as described above, and all three decays were analyzed globally with a three-exponential function. The decays of the integrated fluorescence signals together with the relative amplitudes are presented in Figure 7.

The relative amplitude of the fast component (τ_1_ = 110 fs) increases gradually from 50% to 60% while the intermediate component (τ_2_ = 480 fs) decreases from 40% to 30% upon dilution of the KU dye. This is in excellent agreement with our hypothesis. Hence the fast decay component can be safely attributed to the directly bound KU dye and the intermediate component to the indirectly bound KU dye of the dimer. Although the origin of the longest component is not fully clear, it might originate from loosely bound or free KU dyes in the near zone of the cluster, which decay only partially in the experimental time window of 10 ps.

### Energy Transfer Mechanism

The steady-state luminescence spectra of Au_25_(*p*-MBA)_18_ unambiguously demonstrated an efficient energy transfer process from an excited KU dye to the Au cluster. Furthermore, the two distinct lifetimes observed in the time-resolved fluorescence measurements were attributed to the two individual dye molecules in the bound dimer with donor–acceptor distances of about 1.1 and 1.3 nm. Energy transfer to gold nanoparticles is usually explained by two different mechanisms,^38,39^ Förster resonance energy transfer (FRET) or nanosurface energy transfer (NSET), that show different distance dependencies according to
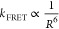
2
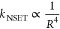
3

Using the separation distances from the MD simulations, the ratio of the rates would equal *k*_1_/*k*_2_ ≈ 2.7 for FRET and 2.0 for NSET. Both values are significantly smaller than the ratio of the observed rates, *k*_1_/*k*_2_ ≈ 4.4. However, due to the 81% energy transfer efficiency, the observed decay times do not perfectly reflect the energy transfer rates, and thus a direct comparison between the values is challenging. Nevertheless, the results are in much better agreement with the FRET mechanism compared to the NSET model. The FRET mechanism has been also demonstrated to be the dominant energy transfer mechanism for small gold nanoparticles at short separation distances,^39^ which is also the case here.

## Conclusion

In summary, we have investigated the energy transfer dynamics from a noncovalently bound KU dye to an atomically precise Au_25_(*p*-MBA)_18_ nanocluster by a combination of experimental and computational methods. The long-lived fluorescence of the KU dye is efficiently quenched in the Au_25_(*p*-MBA)_18_+KU complex, accompanied by an enhancement of the acceptor luminescence. Energy transfer efficiency is determined to be around 81%, suggesting the presence of a competing quenching, presumably an electron transfer process. Interestingly, the luminescence of the Au_25_(*p*-MBA)_18_ clusters is additionally enhanced due to rigidification of the *p*-MBA ligands upon binding of the KU dyes. Time-resolved fluorescence spectra of the complex exhibits two dominant decay components with time constants of 110 ± 20 and 480 ± 70 fs. The two components are attributed to two distinct subpopulations involved in the energy transfer process between the KU dye and the Au_25_(*p*-MBA)_18_. All atomistic MD simulations reveal that the KU dyes can bind to the gold clusters in a monomeric or dimeric form. In the latter, the difference in the center-to-center distances between the individual dyes and the gold cluster is about 0.2 nm, thus explaining the presence of the two distinct populations observed in the time-resolved fluorescence experiment. Moreover, the relative amplitudes depend on the absolute concentration of the KU dye, the faster component increasing in amplitude upon dilution of the dye. The distance dependence of the observed rates supports the FRET mechanism over the NSET mechanism as the dominant energy transfer pathway.

This research gives a detailed, atomistic picture of the dynamics of the energy transfer process in fluorophore-functionalized nanoclusters in solution, along with the surprising observation of the energy transfer of a dimerized fluorophore. At the same time, our research shows that an atomistic understanding of the configuration of the donor–acceptor pairs is required for obtaining a thorough understanding of the overall process.

## Supporting Information Available

All experimental and computational detail; information on materials, methods, and sample preparation, energy transfer efficiency calculation, FLUPS and MD simulation conditions; additional results on UV–vis absorption, fluorescence, FLUPS, MD simulation, and NMR spectra data (PDF)
