# Supplementary material for: Atomistic View of the Energy Transfer in a Fluorophore-Functionalized Gold Nanocluster
Source: J Am Chem Soc. 2023 Jun 28;145(27):14697–704. doi: 10.1021/jacs.3c02292 (PMC10347543; doi:10.1021/jacs.3c02292)
Supplement: Supplementary file 1 — ja3c02292_si_001.pdf [file ja3c02292_si_001.pdf]

Supporting Information

Atomistic View of the Energy Transfer in a Fluorophore-Functionalized Gold Nanocluster

Kyunglim Pyo, María Francisca Matus, Eero Hulkko, Pasi Myllyperkiö, Sami Malola, Tatu Kumpulainen,* Hannu Häkkinen,* Mika Pettersson*

[a]Nanoscience Center, Department of Chemistry, P.O. Box 35, FI-40014, University of Jyväskylä, Finland.

[b]Nanoscience Center, Department of Physics, P.O. Box 35, FI-40014, University of Jyväskylä, Finland.

[c]Nanoscience Center, Department of Biological and Environmental Sciences, P.O. Box 35, FI-40014, University of Jyväskylä, Finland.

*Email: tatu.s.kumpulainen@jyu.fi

*Email: hannu.j.hakkinen@jyu.fi

*Email: mika.j.pettersson@jyu.fi

Table of Contents

Experimental Section.....S1

Figure S1. Representative cross-correlation signal of a well-optimized system for FLUPS.....S3

Figure S2. Molar absorption coefficient of Au_{25}*p*-MBA_{18} and KU emission spectrum.....S5

Figure S3. Absorption spectra of Au_{25}(*p*-MBA)$_{18}$, KU dye and Au_{25}(*p*-MBA)$_{18}$+KU complex.....S5

Figure S4. Luminescence spectra of Au_{25}(*p*-MBA)$_{18}$ upon addition of KU in wavenumbers.....S6

Figure S5. Absorption spectra of Au_{25}(*p*-MBA)$_{18}$+KU complexes for the dual color luminescence S7

Figure S6. Luminescence spectra of Au_{25}(*p*-MBA)$_{18}$+KU with 543 nm and 632 nm excitations.....S8

Figure S7. FLUPS: Integrated time-resolved fluorescence spectra of KU dye and Au_{25}(*p*-MBA)$_{18}$+KU compared with steady-state fluorescence spectrum of KU dye.....S11

Figure S8. Time-resolved fluorescence spectra of free KU dye, and Au_{25}(*p*-MBA)$_{18}$+KU complex with molar ratios of 1:2, 1:1, and 1:0.5.....S12

Figure S9. Fluorescence decays of the reference KU dye and the Au_{25}(*p*-MBA)$_{18}$+KU complexes formed in different molar ratio with three-exponential fits.....S13

Figure S10. ^{1}H NMR spectra of KU dyes in D_2O at different concentration.....S14

Table S1. Concentration effects on ^{1}H chemical shifts of the KU dye.....S15

Figure S11. MD simulation: Simulated systems composed of one Au_{25}(*p*-MBA)$_{18}$ cluster and two KU dyes arranged at 1.3nm, 1.8nm and 2.8nm……………………………………………………………………………………………S16

Figure S12. Distance of the Au_{25}(*p*-MBA)$_{18}$+KU complex as a function of simulation time (1.3 nm) …….S17

Figure S13. Distance of the Au_{25}(*p*-MBA)$_{18}$+KU complex as a function of simulated time (1.8 nm)….…..S18

Figure S14. Distance of the Au_{25}(*p*-MBA)$_{18}$+KU complex as a function of simulated time (2.8 nm)……….S19

Reference………S20

Materials

Deuterium oxide (D_2O, 99.9 atom % D) was purchased from Sigma-Aldrich (Merck) and sodium hydroxide (NaOH, ≥98.5 %) was purchased from VWR Internationals. Water was purified by using a Millipore Elix Essential 3 UV water purification system (15 MΩ·cm). The azaoxotriangulenium (KU) dye was purchased from KU dyes and was used without further modifications. All the chemicals were used as received without further purification.

Methods

UV−vis absorption and photoluminescence spectra of the synthesized gold nanoclusters and KU dye were recorded with Horiba AquaLog spectrophotometer. The UV-vis spectra of the samples upon titration of the Au_{25}(*p*-MBA)$_{18}$ solution with the KU dye solution were recorded with Agilent 8453 UV-vis spectrometer. Sample solutions were measured at ambient conditions using 1 cm and 1 mm path length quartz fluorescence cuvettes from Hellma. Fluorescence emission spectra were measured after exciting the sample at 500 nm wavelength. ^{1}H Nuclear magnetic resonance (NMR) measurements were carried out on Bruker Avance III 500 MHz spectrometer at 30 °C. NMR samples were prepared by dissolving the KU dye in 1 mL D_2O (0.31 mM). The pH of the D_2O solution was adjusted to 10 by adding small drops of 1 M NaOH solution and small amount of the KU sample was continuously added to the D_2O solvent for titration measurement.

The luminescence spectra of the Au_{25}(*p*-MBA)$_{18}$ samples were recorded using a home-built spectrometer consisting of Acton SP2150 spectrograph (grating: 150 grooves / 1000 nm) and a Andor iDus DU490A-1.7 CCD camera. Excitation was achieved with a Helium-Neon laser with lasing wavelengths of 543 and 632 nm placed in a front-face measurement geometry. Typical excitation power was about 1–2 mW. The luminescence from the sample was collected using a pair of focusing lenses and focused onto the entrance slit of the spectrograph. Scattered excitation light was removed by a long-pass filter (KX-19) placed in front of the entrance slit of the spectrograph. Due to the low signal, the slits were fully open (2 mm) during the experiments. The spectral sensitivity of the detection system was determined by measuring the emission spectrum of a NIST traceable Tungsten halogen lamp. Background measured from the aqueous solvent was subtracted from all measured spectra and the spectra were subsequently corrected for the spectral sensitivity.

Synthesis of Au_{25}(*p*-MBA)$_{18}$

$Au_{25}(p\text{-}MBA)_{18}$ clusters were synthesized according to the procedure described elsewhere.[1]

Steady-state fluorescence measurement of KU dye upon addition of $Au_{25}(p\text{-}MBA)_{18}$

1 mL of 0.18 mM $Au_{25}(p\text{-}MBA)_{18}$ and 1.5 mL of 0.0217 mM KU dye was prepared in pH 10 aqueous solution. The prepared KU dye solution was transferred to the 1 cm quartz cuvette and 5 µL of $Au_{25}(p\text{-}MBA)_{18}$ solution was carefully added to the KU solution. After thoroughly mixing the solution with a glass pipette, the fluorescence was measured by exciting the sample at 500 nm wavelength. 5 µL of $Au_{25}(p\text{-}MBA)_{18}$ solution was added until the fluorescence of the KU dye was completely quenched, which was 75 µL in total for this experiment. The molar ratio of the $Au_{25}(p\text{-}MBA)_{18}$ and the KU dye was calculated based on the molar absorption coefficients and added volumes. It is good to note that, the KU dye absorbance was limited to 0.2 abs (at 530 nm, 0.0217 mM) to avoid the inner-filter effect from the highly concentrated solution. Moreover, to avoid the KU dye solution from getting diluted by the added $Au_{25}(p\text{-}MBA)_{18}$ solution, the $Au_{25}(p\text{-}MBA)_{18}$ solution was prepared in high concentration.

Steady-state luminescence measurement of $Au_{25}(p\text{-}MBA)_{18}$ upon addition of the KU dye

1.0 or 1.5 mL of dilute (c = 5.8 µM) $Au_{25}(p\text{-}MBA)_{18}$ solution was prepared in pH 11 aqueous solution. Another solution with high KU dye concentration (0.2–0.5 mM) and an equivalent concentration of $Au_{25}(p\text{-}MBA)_{18}$ at the same pH was prepared separately. The dilute $Au_{25}(p\text{-}MBA)_{18}$ solution was transferred to a 1 cm quartz cuvette and the luminescence was collected by exciting the sample at 543 nm or 632 nm wavelength. A small volume (5 to 30 µL) of the KU dye solution was added to the cuvette. After thoroughly mixing the solution with a glass pipette, the luminescence was measured again. The additions were continued until no further luminescence enhancement was observed. This occurred above molar ratio of $[mol_{KU}]/[mol_{Au25}]$ = 5. Concentrations and molar ratios were estimated from the molar absorption coefficients and added volumes of the stock solutions.

Preparation of $Au_{25}(p\text{-}MBA)_{18}$ + KU dye complex solution for fluorescence up-conversion spectroscopy (FLUPS) measurement

The concentration for the materials were calculated by using the extinction coefficient value.[1] All FLUPS spectra were measured using a quartz cuvette with 1 mm optical pathlength. 1 mL of 0.43 mM KU dye (A = 0.4 at 530 nm) and 1 mL of 0.22 mM $Au_{25}(p\text{-}MBA)_{18}$ (A = 0.15 at 700 nm) were prepared in aqueous solution at pH 11. Then, 0.5 mL of each solution were combined to yield the complex with the ratio of $[Au_{25}]$:[KU] = 1:2 right before the measurement. For the control experiment ($[Au_{25}]$:[KU]= 1:1 and 1:0.5), the concentration of the $Au_{25}(p\text{-}MBA)_{18}$ solution was fixed to 0.22 mM and the concentration of the KU dye was adjusted accordingly.

Fluorescence up-conversion spectroscopy (FLUPS)

Broadband FLUPS measurements were performed on an in-house assembled setup (assembled from an assembly kit supplied by LIOP-TEC GmbH) similar to those described in detail in refs. 2–4. The FLUPS setup utilizes a standard 1 kHz Ti:Sapphire amplified system (Coherent Astrella) producing 90 fs pulses at 800 nm as the main laser source. Excitation at 520 nm was achieved by using an output from an in-house assembled two-stage noncollinear optical parametric amplifier (NOPA) pumped with the amplified laser system.[5] The mode and size of the excitation pulses were adjusted by using a telescope equipped with a 100 μm pinhole at the focus and subsequently compressed using a prism compressor to minimize the cross-correlation signal between the pump and the gate pulses. Polarization and intensity of the excitation pulses were controlled by using a combination of a wire-grid polarizer and a half-waveplate. All measurements were performed at magic angle (54.7°) polarization with an excitation power of ca. 0.3 mW (300 nJ/pulse). The excitation pulses were focused down to a 100 μm spot at sample resulting in an excitation intensity of ca. 1 mJ/cm^2. The gate pulses at 1345 nm were produced by an in-house assembled optical parametric amplifier and subsequently compressed by using a prism compressor.[6] Time-resolution of the setup was estimated from the cross-correlation signal between the pump and gate pulses. The value is reported as a full-width at half-maximum (FWHM) of the integrated cross-correlation signal fitted with a Gaussian function. The FWHM of the cross-correlation was sensitive to the compression of both the excitation and gate pulses and varied between 120 and 140 fs depending on the optimization of the overall setup. At optimal compression, the integrated cross-correlation signal was nearly indistinguishable from the cross-correlation signal monitored at a single wavelength (see Figure S1 for a representative cross-correlation signal of a well-optimized system).

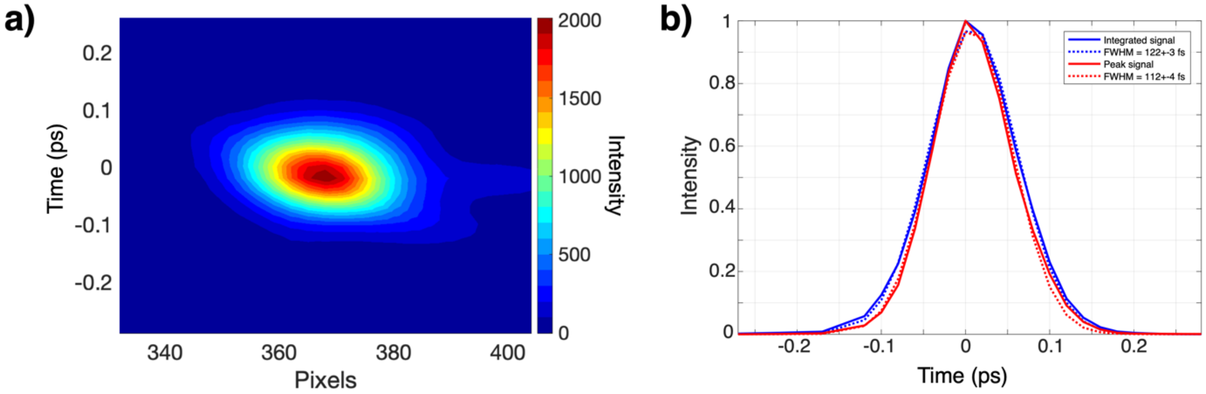

Figure S1. a) A representative cross-correlation (sum frequency) signal between the pump and gate pulses. The signal is presented as a function of the CCD pixel number. b) Time profiles of the integrated (blue) and peak (red) cross-correlation signals (solid lines) together with Gaussian fits (dashed lines). The full-width at half-maximum (FWHM) values of the Gaussian functions are given in the legend.

Detection of the up-converted signal was performed with a home-built spectrograph coupled to a CCD camera (Andor, DV420A-BU). Scattered excitation light was suppressed by the use of a 550 nm long-pass filter, which limited the detection range to ca. 560 nm. Time-resolved fluorescence spectra were recorded from –2 ps up to 10 (complexes) or 20 ps (free KU dye). Due to the relatively low fluorescence signal, measurements consisted of 20–30 successive scans with 1 s collection time at each time step. All presented spectra represent the average intensity from the successive scans. Averaged spectra were transferred into spectra vs. wavelength and subsequently photometrically corrected by a calibration with secondary emissive standards as described in ref. 2. The chirp polynomial due to group velocity dispersion

was determined by monitoring the instantaneous response of the reference KU dye sample in the same solvent system. The polynomial function was then used to remove the chirp from the experimental spectra of the Au_{25}(*p*-MBA)$_{18}$+KU complexes. The samples were continuously stirred during the measurements by bubbling with nitrogen next to the excitation spot. Absorption spectra of the samples were recorded before and after the experiments. No changes were observed in the absorption spectra indicating that no photodegradation took place during the experiment.

Model structure of Au_{25}(*p*-MBA)$_{18}$+KU complex and molecular dynamics (MD) simulations

A 100-ns equilibrated model of fully deprotonated Au_{25}(*p*-MBA)$_{18}$ cluster was obtained from a previous study.[1] The nanocluster was combined with two KU dyes and solvated in a periodic cubic box of water with 0.15 M NaCl to neutralize the systems. Three different starting configurations were evaluated, where the KU dyes were arranged at a) 1.3 nm, b) 1.8 nm, or c) 2.8 nm away from the center of mass of the nanocluster (**Figure S11**). In the first configuration, a T-stacking interaction between the KU dyes and three stacked *p*-MBAs was induced manually in order to represent the potential association of the dyes with the highly-ordered π–π stacked groups observed in solution for the isolated Au_{25}(*p*-MBA)$_{18}$.[1] All molecular dynamics (MD) simulations were performed with GROMACS 2022[7] using a previously published force field for thiolate-protected gold nanoclusters.[8] Energy minimization of each system were performed using the steepest descent method followed by a short equilibration consisting of 10 ns NVT (constant number of particles, volume, and temperature) at 300 K followed by 10 ns NPT (constant number of particles, pressure, and temperature) at 300 K and 1 bar pressure using the V-rescale thermostat and Berendsen barostat.[9] Then, 500 ns of production MD was carried out by keeping the temperature at 300 K with the velocity-rescale thermostat[10] and pressure at 1 bar using Parinello-Rahman barostat[11] with a period of 2.0 ps. The leapfrog Verlet integrator with a 2.0 fs timestep was used. The van der Waals interactions were modeled with Lennard–Jones potentials truncated at 1.0 nm, while electrostatic interactions were modeled with the particle-mesh Ewald (PME) method[12] with a cutoff of 1.0 nm and 0.12 nm grid spacing. For improved performance, bond lengths to hydrogens in the nanocluster were constrained with the LINCS algorithm.[13] The visualization of trajectories and the distance analyses of the Au_{25}(*p*-MBA)$_{18}$+KU complex as a function of simulated time (**Figure S12-S14**) were carried out in VMD.[14]

Molar absorption coefficient of $Au_{25}(p\text{-MBA})_{18}$ and fluorescence spectrum of KU

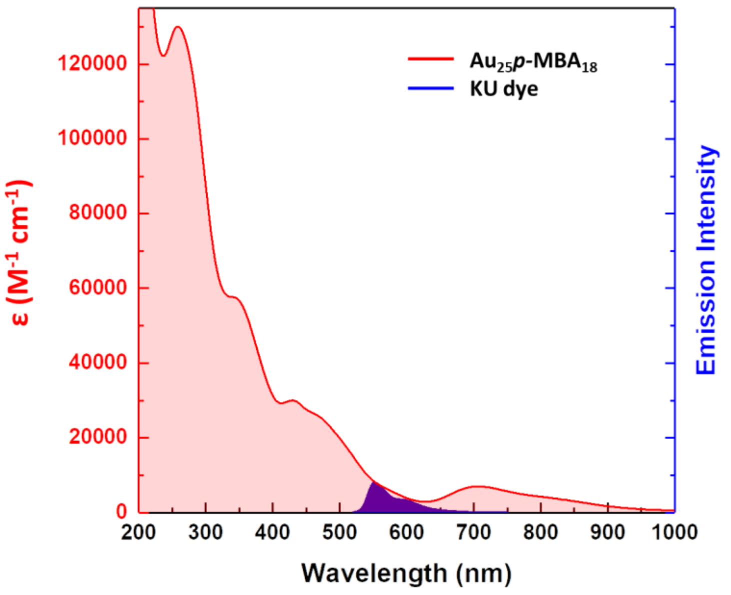

Figure S2. Molar absorption coefficient of $Au_{25}(p\text{-MBA})_{18}$ and KU emission spectrum used in the energy transfer calculation. The emission was observed after exciting at 500 nm wavelength.

Steady-state absorption spectra of $Au_{25}(p\text{-MBA})_{18}$+KU complex

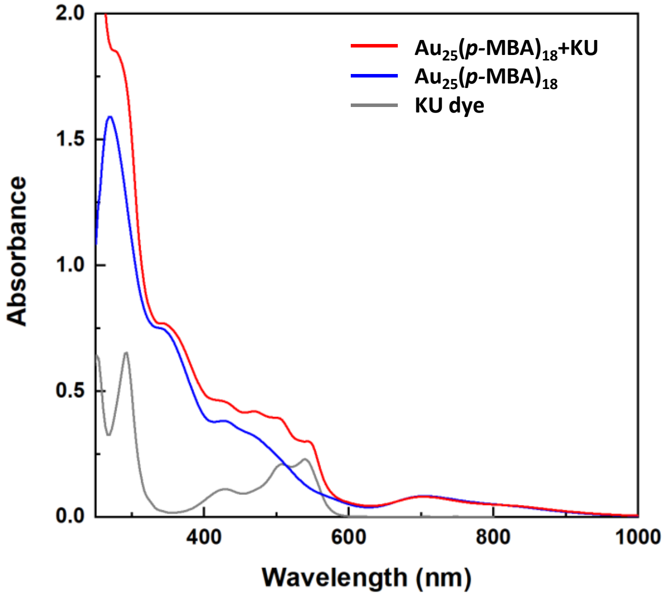

Figure S3. Absorption spectra of $Au_{25}(p\text{-MBA})_{18}$, KU dye and $Au_{25}(p\text{-MBA})_{18}$+KU complex with 1:2 molar ratio ($[mol_{Au}]/[mol_{KU}]$).

Steady-state luminescence spectra of Au_{25}(*p*-MBA)$_{18}$ upon addition of KU

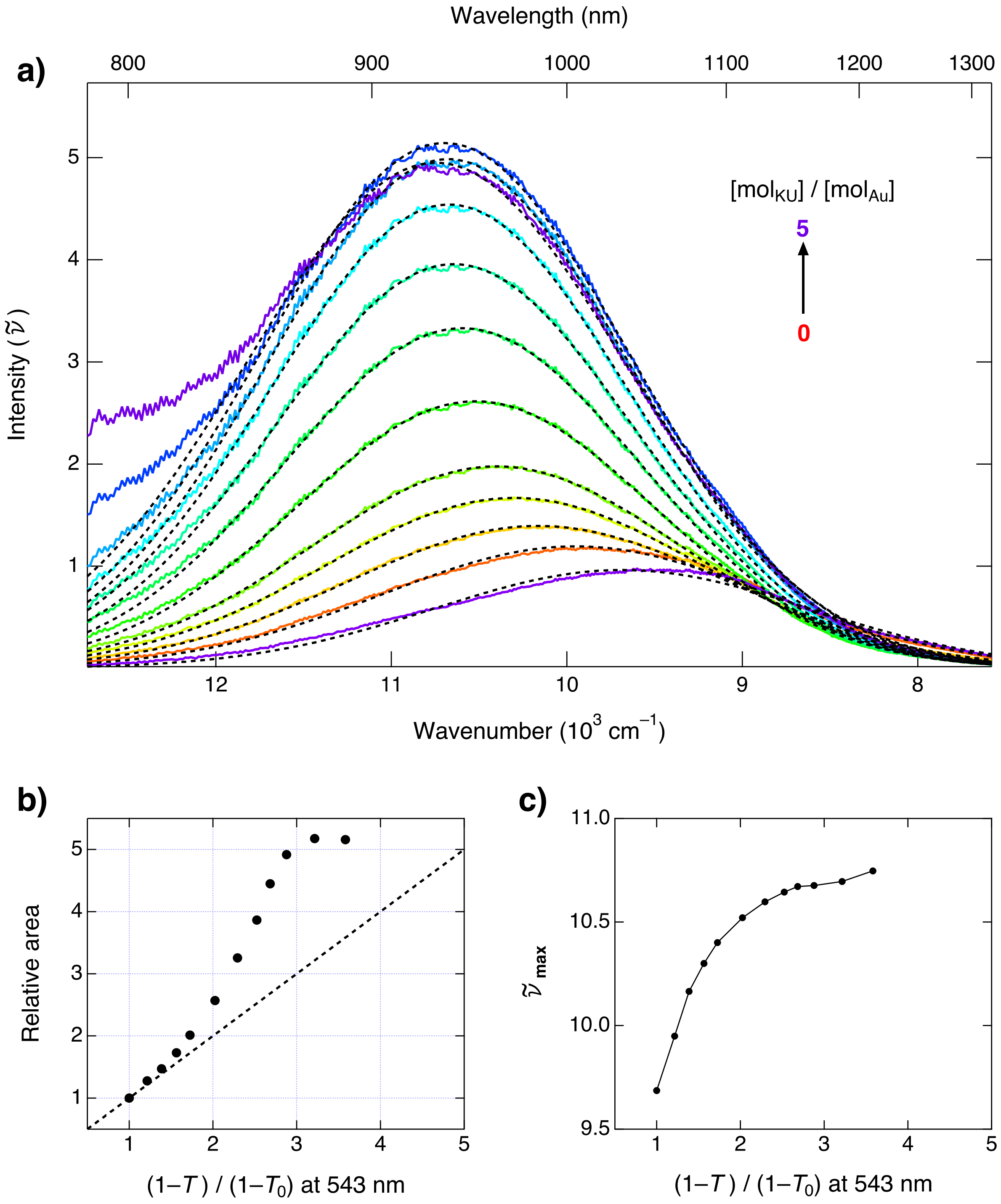

Figure S4. a) Steady-state luminescence spectra of Au_{25}(*p*-MBA)$_{18}$ (c = 5.8 μM) in wavenumber domain upon addition of the KU dye solution. The black dashed lines represent fits with a Gaussian band-shape function. b) Relative Gaussian-band areas from the fits in a) as a function of relative absorbance at the excitation wavelength. c) Band maxima of the Gaussian band-shapes as a function of relative absorbance at the excitation wavelength.

Determining the energy-transfer efficiency from KU to Au_{25}

Quantitative determination of the energy-transfer efficiency was complicated by the additional luminescence enhancement that was attributed to the rigidifying effect of the ligands upon association of the KU dye (**Figure 2** and discussion in the main text). Therefore, we performed a comparative measurement where the luminescence spectra were recorded from the same sample at two excitation wavelengths of 543 nm and 632 nm upon the addition of the KU dye. Both excitation beams were overlapped at the sample using the same angle of incidence (AOI $\approx$ 32°) to maintain identical measurement geometry. Due to the limited space available in the home-built spectrometer (adapted from another setup), the measurement conditions (geometry, excitation power) were slightly different from the first measurement presented in **Figure 2** of the main text and thus, the results might not be directly comparable. Inner filter effects, in particular, would be expected to depend on the exact measurement geometry. Due to these reasons, measurements were performed on a rather dilute solutions where the magnitude of the inner filter effects is expected to be small.

The overall changes observed in the absorption spectra of the sample upon addition of KU dye prevented direct comparison of the relative luminescence intensities at the two excitation wavelengths. The changes in the long-wavelength region above 600 nm were particularly puzzling and at first glance seemed like the overall increase that could be caused by scattering. However, scattering should be clearly visible in the short wavelength region, which is not the case here. Therefore, the changes are attributed to real changes in the absorption spectrum of the sample. The absorption spectra of Au_{25}(*p*-MBA)$_{18}$ upon addition of the KU dye in the full wavelength region are presented in **Figure S5**. The long wavelength region is magnified in the inset where the two excitation wavelengths are highlighted by the green and red arrows.

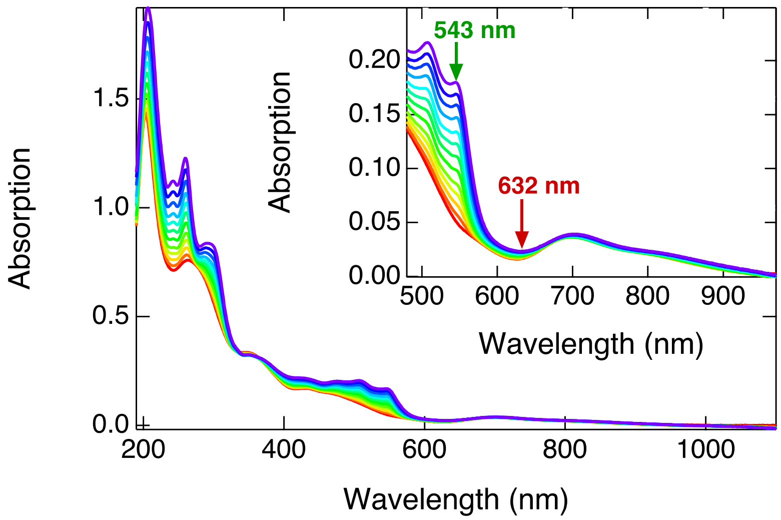

Figure S5. Absorption spectra of Au_{25}(*p*-MBA)$_{18}$ (*c* = 5.8 μM) upon addition of increasing concentration of the KU dye up to about 24 μM.

Although the absorption changes minimally at 632 nm, the relative change is rather large (50%) due to the very small absorbance of the sample at this wavelength. Therefore, we had to take this change into account. Secondly, since the absorption of the KU dye at this wavelength is effectively zero (see **Figure**

S3), we assumed that the 632 nm light selectively excites Au_{25}(*p*-MBA)$_{18}$. This allowed us to calculate the relative luminescence quantum yields of Au_{25}(*p*-MBA)$_{18}$ at each KU concentration, which is required for the determination of the energy-transfer efficiency. The relative quantum yields were determined according to the protocol reported in ref. 15. The luminescence areas were extracted by integrating the corrected and background subtracted spectra over the whole detection range in wavelength according to:

$$F = \int I(\lambda) d\lambda \quad (1)$$

The absorptance values, *f*, of the samples were calculated from the absorption values at the excitation wavelengths of 632 nm indicated in **Figure S5** according to:

$$f = 1 - 10^{-A(\lambda_{\mathrm{ex}})} \quad (2)$$

The luminescence spectrum measured in the absence of the KU dye was considered as the reference and the quantum yields in the presence of KU were determined relative to this value according to:

$$\frac{\Phi_{\mathrm{Au}}}{\Phi_{\mathrm{ref}}} = \frac{F_{\mathrm{Au}}}{F_{\mathrm{ref}}} \cdot \frac{f_{\mathrm{ref}}}{f_{\mathrm{Au}}} \cdot \frac{n_{\mathrm{Au}}^2}{n_{\mathrm{ref}}^2} \quad (3)$$

In eq. (3), the values with subscript **ref**, are the values in the absence of the KU dye, and values with subscript **Au** are the values at each KU concentration. The last term related to the refractive indices can be ignored because same solvent system was used for both samples. The luminescence spectra of Au_{25}(*p*-MBA)$_{18}$ upon increasing concentration of the KU dye at the two excitation wavelengths are presented in **Figure S6**. The relative quantum yield of Au_{25}(*p*-MBA)$_{18}$ upon 632 nm excitation, a function of the molar ratio [mol_{KU}]:[mol_{Au}] is given in the inset of b).

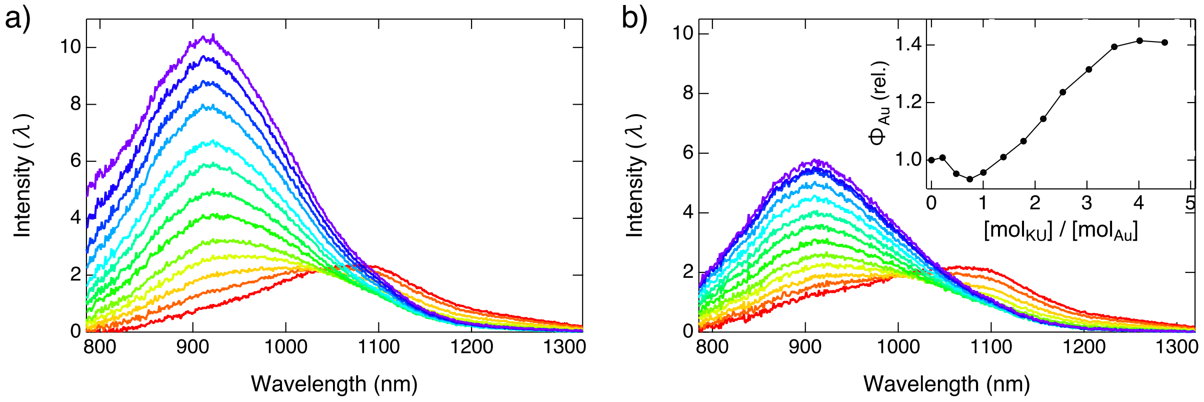

Figure S6. Luminescence spectra of Au_{25}(*p*-MBA)$_{18}$ (*c* = 5.8 µM) upon addition of increasing concentration of the KU dye upon a) 543 nm and b) 632 nm excitation. The relative luminescence quantum yield of Au_{25}(*p*-MBA)$_{18}$ upon 643 nm excitation as a function of the molar ratio is given in the inset.

The quantum yield of Au_{25}(*p*-MBA)$_{18}$ increases by about 41% upon addition of 4 to 5 equivalents of KU dye, after which no further enhancement is observed. At this point, the fluorescence of the free KU dye starts to appear in the spectra excited at 543 nm (**Figure 2** in the main text).

Since the observed luminescence in the Au_{25}(*p*-MBA)$_{18}$+KU complexes originates solely from the Au_{25}(*p*-MBA)$_{18}$ clusters, the total luminescence intensity upon 543 nm excitation can be expressed in

terms of the relative quantum yield of the clusters determined above. However, since both the $Au_{25}(p\text{-MBA})_{18}$ and the KU dye show significant absorption at 543 nm, both the direct and indirect excitations must be considered. Thus we need to determine the relative absorption probabilities of the $Au_{25}(p\text{-MBA})_{18}$ and the KU dye in the complex at each KU concentration. Due to the additive nature of Beer-Lambert law, the amount of absorbed light in an infinitely thin slice along the light path is a linear sum of the two contributions. Thus, the amount of light absorbed by each component in the whole sample is directly related to the relative absorbance of the components. Assuming that absorption of $Au_{25}(p\text{-MBA})_{18}$ remains constant at 543 nm upon addition of the KU dye, the relative absorption probabilities can be calculated according to:

$$P_{\mathrm{Au}} = \frac{A_{\mathrm{Au}}}{A_{\mathrm{tot}}} \tag{4}$$

$$P_{\mathrm{KU}} = \frac{A_{\mathrm{tot}} - A_{\mathrm{Au}}}{A_{\mathrm{tot}}} \tag{5}$$

where A_{Au} is the absorbance before addition of KU and A_{tot} the total absorbance of the $Au_{25}(p\text{-MBA})_{18}$+KU complex sample. The total luminescence intensity can be now expressed as a sum of the two contributions, each dependent on the total absorptance scaled by the absorption probabilities and the relative quantum yields. In addition, the luminescence due to indirect excitation depends on the energy-transfer efficiency. The total luminescence yield can be thus calculated according to:

$$F_{\mathrm{Au:KU}} = \int I_{\mathrm{Au:KU}}(\lambda) d\lambda = (f_{\mathrm{Au}}\Phi_{\mathrm{Au}} + f_{\mathrm{KU}}\Phi_{\mathrm{Au}}\Phi_{\mathrm{ET}}) \cdot f_{\mathrm{scaling}} \tag{6}$$

where f_{Au} and f_{KU} are the total absorptance values scaled by the relative absorption probabilities at 543 nm, Φ_{Au} the relative quantum yields determined above, and f_{scaling} is an instrument related scaling parameter. The scaling parameter reflects the relative intensity that should be obtained at the corresponding absorptance value and can be calculated from the first recorded spectrum in the absence of the KU dye according to:

$$f_{\mathrm{scaling}} = \frac{F_0}{f_{\mathrm{Au}}} \tag{7}$$

Here F_0 is the integrated luminescence intensity upon 543 nm excitation before addition of the KU dye and f_{Au} is the corresponding absorptance value at 543 nm. With eq. (7), the total luminescence yield at each KU concentration can be calculated from the absorption spectra and the luminescence spectrum measured in the absence of the KU dye with 543 nm excitation. The measured and estimated total luminescence yields are presented in **Figure 3** of the main text. Optimal energy transfer rate was found by minimizing the relative error at each measurement point yielding Φ_{ET} = 81%. The agreement between the measured and estimated total luminescence yields is excellent (relative error <2.1%) up to $[\mathrm{mol_{KU}}]:[\mathrm{mol_{Au}}]$ = 3.5. Above this ratio, the luminescence enhancement begins to plateau and the fluorescence of the free KU dye starts to grow in slowly increasing the observed luminescence yield.

Fluorescence spectra Au_{25}(*p*-MBA)$_{18}$+KU complex

Presumably, all fluorescence detected in the FLUPS measurements originated from the KU dye. To verify this, we compared the time-integrated fluorescence spectra from FLUPS to the steady-state fluorescence of the KU dye. In order to account for the long-pass filter (LP550), used to suppress the scattered excitation light in the FLUPS measurements, the steady-state fluorescence spectrum was multiplied by the transmission profile of the LP550 filter (**Figure S7a**). The FLUPS spectra were integrated from 0.2 ps up to 20 ps for the reference KU sample and from 0.2 ps up to 5 ps for the Au_{25}(*p*-MBA)$_{18}$+KU complexes. Comparison between the integrated and steady-state fluorescence spectra are presented in **Figures S7b** and **S7c**. The concentrated samples with [KU] = 0.2 mM (Figure S4b) show a significantly smaller fluorescence intensity at 550–570 nm in the time-integrated FLUPS spectra than in the steady-state spectrum measured from a diluted sample. However, the time-integrated spectra of the reference KU sample (black in **Figure S7b**) and the Au_{25}(*p*-MBA)$_{18}$+KU complex with 1:2 molar ratio (green in **Figure S7b**) with identical concentration of the KU dye are very similar. On the other hand, the time-integrated fluorescence spectra of the Au_{25}(*p*-MBA)$_{18}$+KU complexes with 1:1 and 1:0.5 molar ratios are almost identical to the steady-state fluorescence spectrum. This suggest that the difference observed in the spectra of the samples with higher KU concentration is likely due to inner filter effect (reabsorption). This interpretation is also supported by the absorption spectrum of the KU dye that extends approximately up to 580 nm (**Figure S3**). Thus, the results unambiguously demonstrate that the time-resolved fluorescence observed in the FLUPS spectra originate solely from the KU dye.

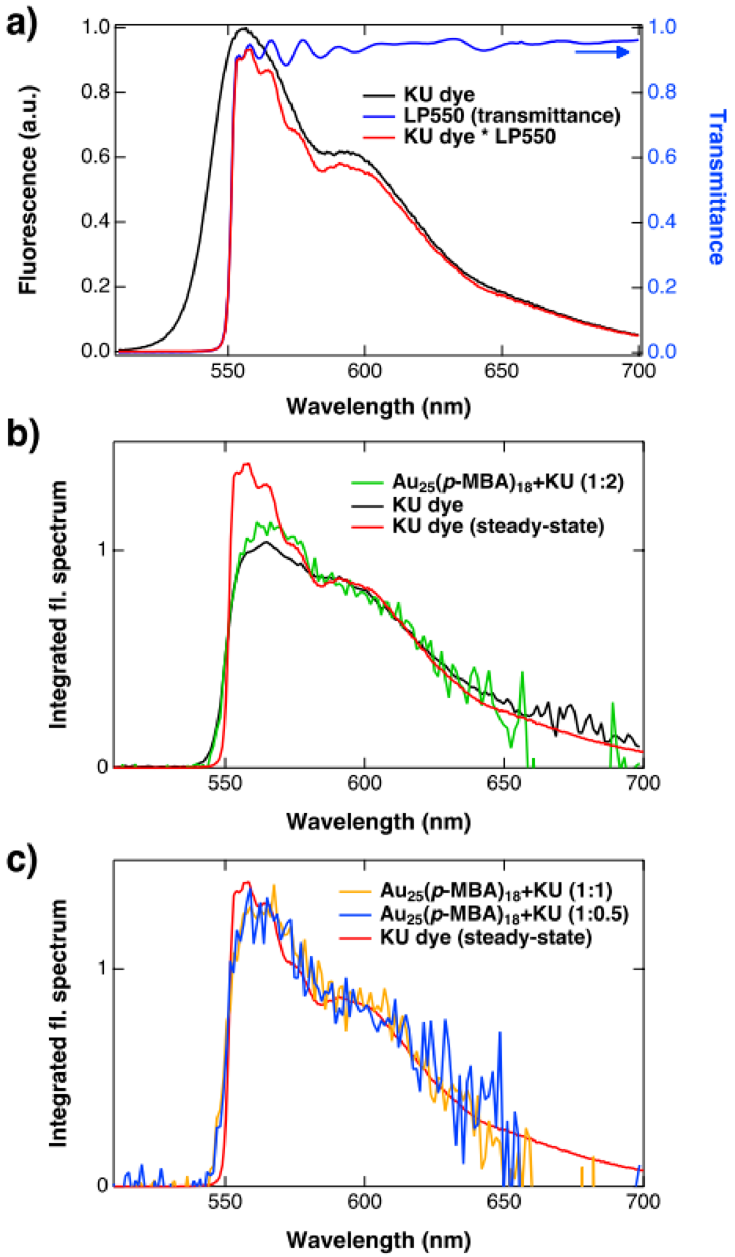

Figure S7. a) Steady-state fluorescence spectrum of a diluted KU dye solution (black), transmission spectrum of the LP550 longpass filter (blue) and the steady-state fluorescence spectrum multiplied by the LP550 transmission profile. b) Integrated time-resolved fluorescence spectra of the free KU dye (black) and the Au_{25}(*p*-MBA)$_{18}$+KU complex with 1:2 molar ratio (green) overlaid with the steady-state fluorescence spectrum corrected for the transmission of the LP550 filter (red). c) The same comparison for the Au_{25}(*p*-MBA)$_{18}$+KU complexes with 1:1 and 1:0.5 molar ratios corresponding to a 2-fold and 4-fold lower concentration of the KU dye as compared to 1:2 molar ratio. The time-integration was performed from 0.2 ps up to 20 ps and 5 ps for the free KU dye and the Au_{25}(*p*-MBA)$_{18}$+KU complexes, respectively.

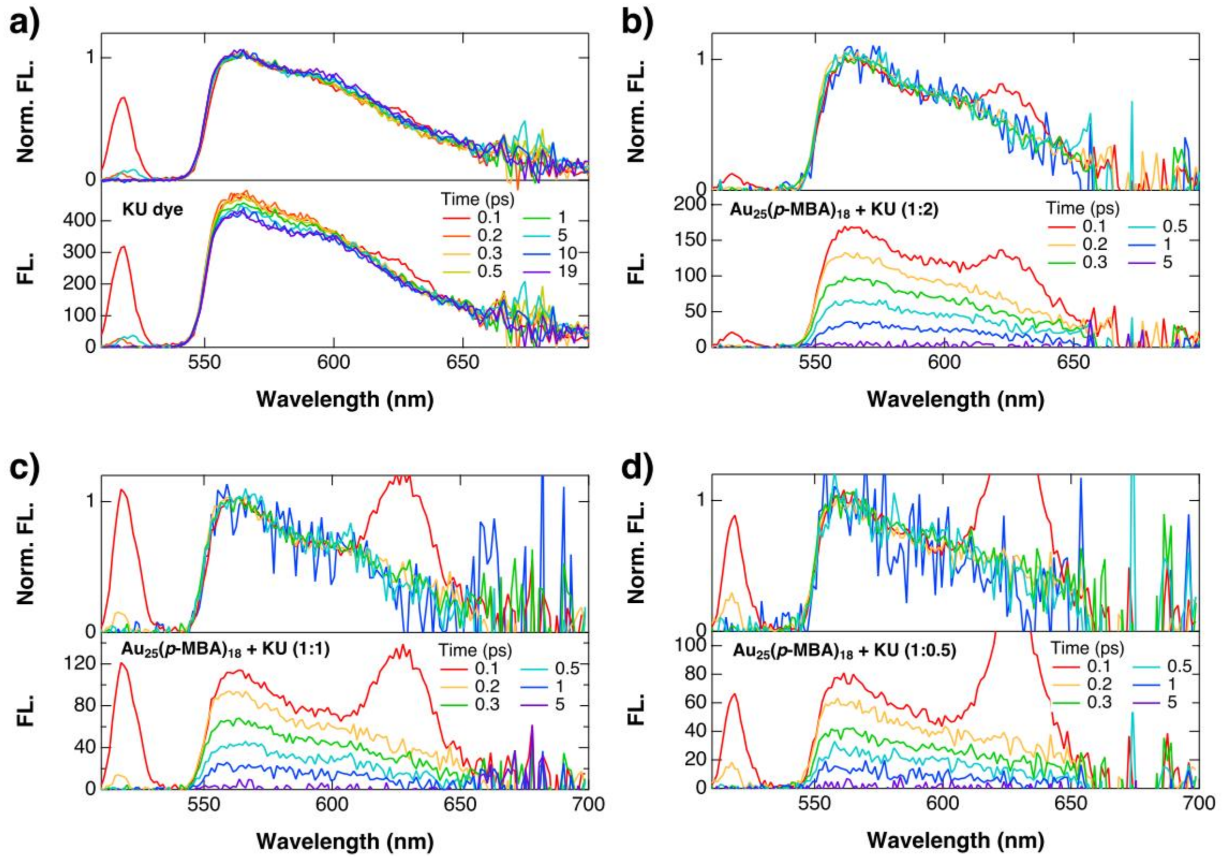

Figure S8. Time-resolved fluorescence spectra of a) free KU dye, and Au_{25}(*p*-MBA)$_{18}$+KU complex with molar ratios of b) 1:2, c) 1:1, and d) 1:0.5 at selected time points. The top panels show the normalized spectra. The spectrum measured at 5 ps is omitted from the top panels of b)–d) for clarity. Please, note the different time points in a).

The fluorescence decays were extracted by integrating the total fluorescence signal in the range of 560–610 nm for the Au_{25}(*p*-MBA)$_{18}$+KU complexes and 560–590 nm for the reference KU sample. Integrating the signal significantly improved the signal to noise ratio. The decay times were obtained by fitting the decays with a three-exponential function convolved with a Gaussian simulated instrument response function (IRF). This allowed us to reliably resolve lifetimes that were somewhat shorter than the FWHM of the measured cross-correlation function. Decay time of the fast component (ca. 100 fs) observed for the Au_{25}(*p*-MBA)$_{18}$+KU complexes was reproduced in all experiments irrespective of the time resolution of the overall setup that varied in the range of 120-140 fs (FWHM of the integrated cross-correlation signal). However, the relative amplitude of the fast component was found to be somewhat sensitive to the time resolution complicating a detailed comparison between data measured on different days. Therefore, the comparative measurements presented in the main text were measured in a single session with identical experimental conditions. Furthermore, the decays of the Au_{25}(*p*-MBA)$_{18}$+KU complexes at different molar ratios were analyzed globally with a three-exponential function where the decay times were the same for all three decays, but amplitudes were allowed to vary freely. This enabled a highly reliable comparison of the relative amplitudes between the different samples. The decay times of the Au_{25}(*p*-MBA)$_{18}$+KU complexes reported in the main text correspond to the results obtained from the global

fit of the three decays. The associated errors represent the 95% confidence intervals obtained from the nonlinear fit. However, it should be noted that measurements performed on different days with slightly different experimental conditions yielded decay times within the 95% confidence intervals. The decays and exponential fits of all samples are presented in **Figure S9**. The long-lived component of the reference KU sample was constrained to 19 ns as determined from time-correlated single photon counting. The decay times, errors and amplitudes are reported in the main text.

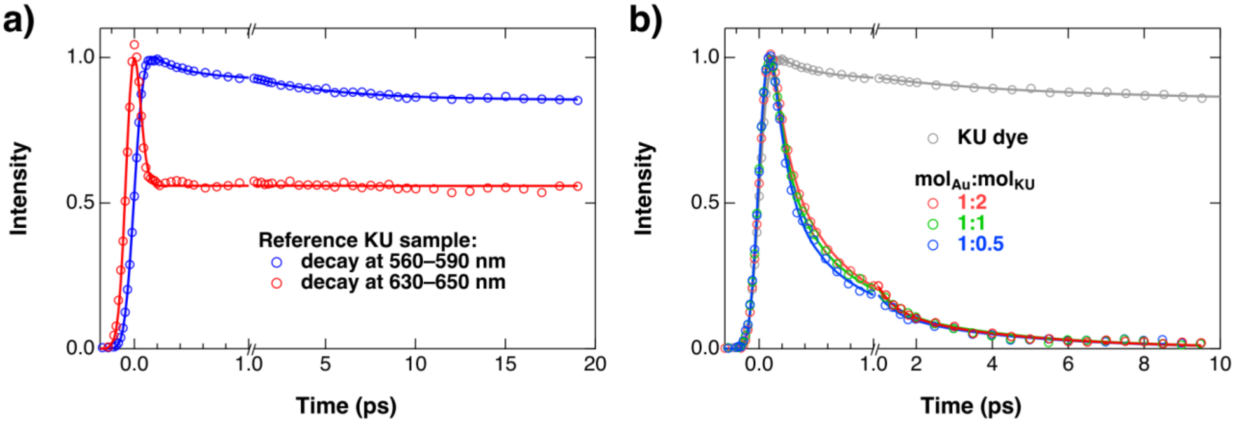

Figure S9. a) Fluorescence decays (markers) of the reference KU sample at the high- and low-energy sides of the fluorescence band. Integration ranges are given in the legend. The lines represent fits with a two- (630–650 nm) and three-exponential (560–590 nm) functions. The sharp peak a *t* = 0 ps observed in the decay at 630–650 nm is due to the Raman scattering peak. b) Fluorescence decays (markers) of the reference KU sample and the Au_{25}(*p*-MBA)$_{18}$+KU complexes together with three-exponential fits (lines). The fits of the complexes correspond to the global fit of all three decays simultaneously as explained in the text. The decays represent the integrated fluorescence signal between 560–610 nm for the complexes and between 560–590 nm for the reference KU sample. The long-lived dominant component of the reference KU sample was constrained to 19 ns in all fits.

Concentration dependent ^{1}H-NMR spectra and chemical shifts of the KU dye

As shown in **Figure S10**, all the aromatic protons (7.2-8.3 ppm) shift upfield as the concentration increases. Note that the highest concentration for the NMR measurement was fixed at 0.21 mM, the same concentration that was used in the FLUPS measurement. The maximum shift occurrs for H_b and H_c protons (Δppm~0.1 ppm) followed by H_e, H_d, and H_a protons which shift upfield by Δppm ~0.06 and 0.05 ppm, respectively. A similar proton upfield shift are observed for anthracycline, coralyne, and other aromatic dyes in which the degree of aggregation was found to be affected by the solvent polarity, temperature, and concentration. [16-20] In the higher aggregating conditions, the 1H NMR peaks for the aromatic protons shift upfield by the effect of the ring current and the shielding effect of the stacked dyes. Therefore, these upfield shifts of the KU aromatic protons can be attributed to the formation of dimers or higher-order aggregates as predicted from the FLUPS. By further investigation, the KU behavior of forming aggregates is once more agreed by the MD simulation results. (**Figure S14**).

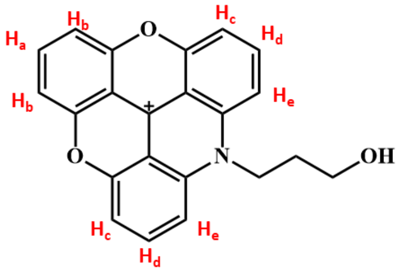

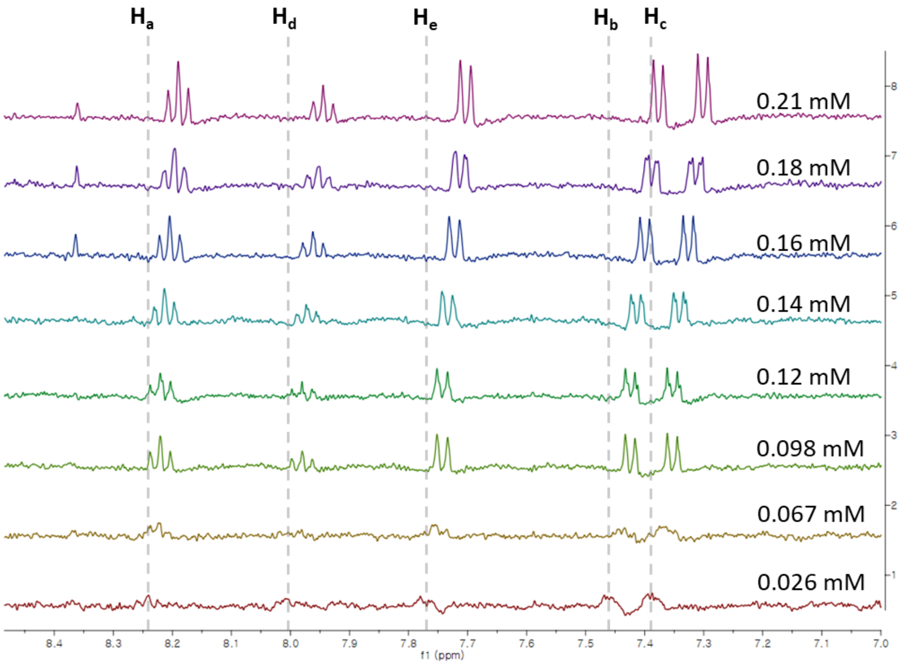

Figure S10. ^{1}H NMR spectra of the aromatic protons of the KU dyes in D_2O at different concentrations in range 0.026 - 0.21 mM at 30°C and pH 11. Chemical shift values of the protons for 0.026 and 0.21 mM are listed in **Table S1**.

Table S1. Concentration effects on chemical shifts of protons listed in Figure S8.

	H_a *(ppm)*	H_d *(ppm)*	H_e *(ppm)*	H_b *(ppm)*	H_c *(ppm)*
**0.026 mM**	8.24	8	7.77	7.47	7.4
**0.21 mM**	8.19	7.94	7.71	7.37	7.3
Δppm	0.05	0.06	0.06	0.1	0.1

Supplementary figures from MD simulations

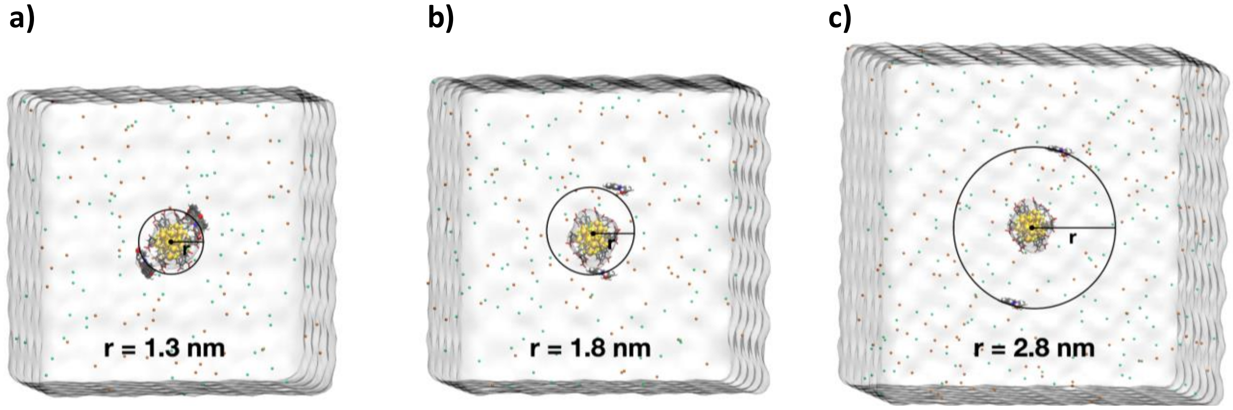

Figure S11. Simulated systems composed of one Au_{25}(*p*-MBA)$_{18}$ cluster and two KU dyes arranged at a) 1.3 nm, b) 1.8 nm, or c) 2.8 nm away from the center of mass of the cluster, surrounded by ions (0.15 M NaCl; Na^+ and Cl^- displayed in green and orange spheres, respectively) in a water solvent box. The box volume of configurations a), b), and c) are 773.62 nm^3, 982.11 nm^3, and 1680.91 nm^3, respectively.

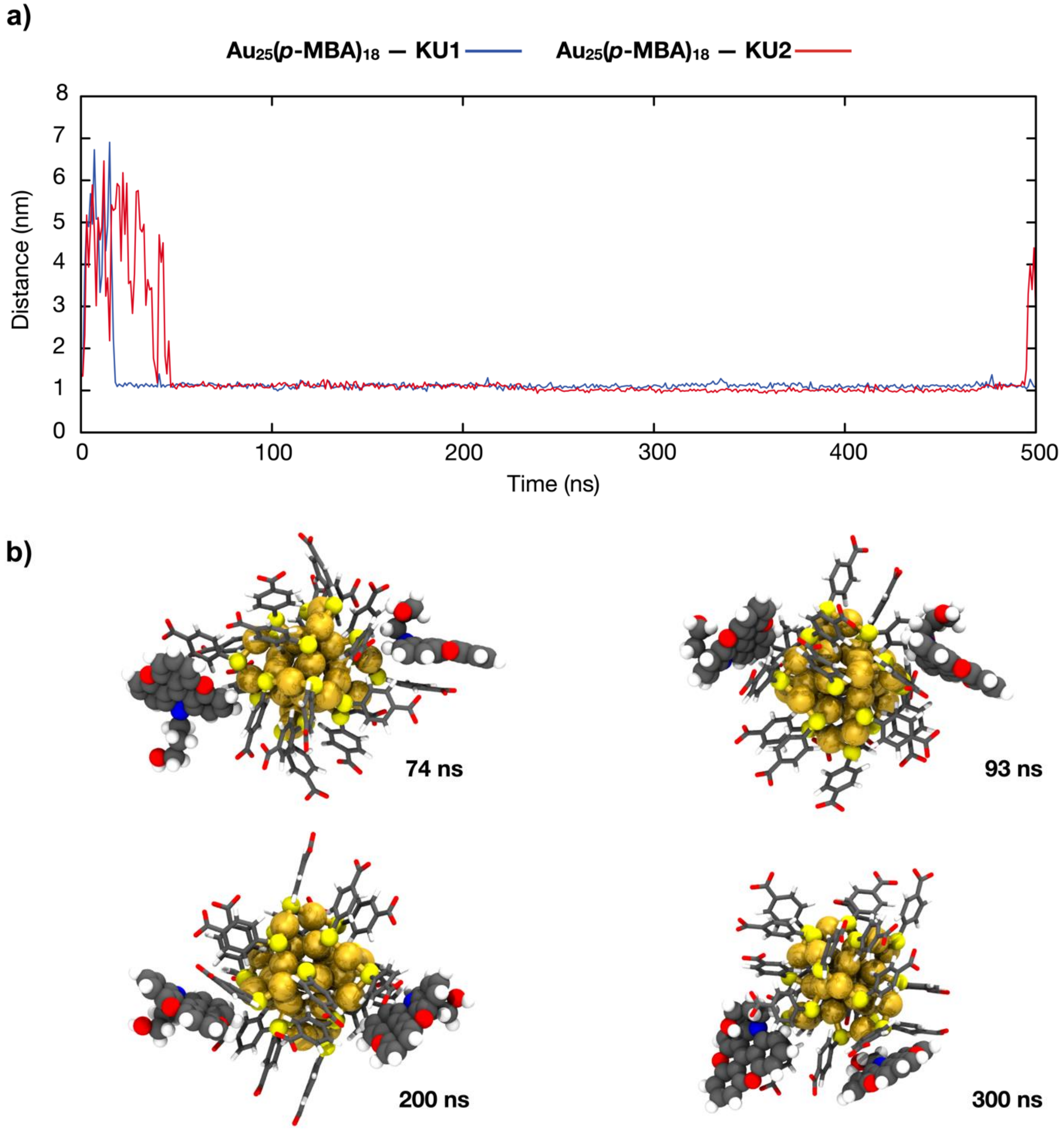

Figure S12. Distance analysis of the Au_{25}(p-MBA)$_{18}$+KU complex as a function of simulated time when KU dyes are placed at 1.3 nm with respect to the center of mass of Au_{25}(p-MBA)$_{18}$ cluster. (a) distance between the Au_{25}(p-MBA)$_{18}$ cluster and each KU dye monitored over the entire 500-ns MD trajectory. (b) representative snapshots extracted from the MD trajectory (time interval: 50-490 ns) when both KU dyes interact with the *p*-MBA ligands as a monomer and remain equidistant from the nanocluster.

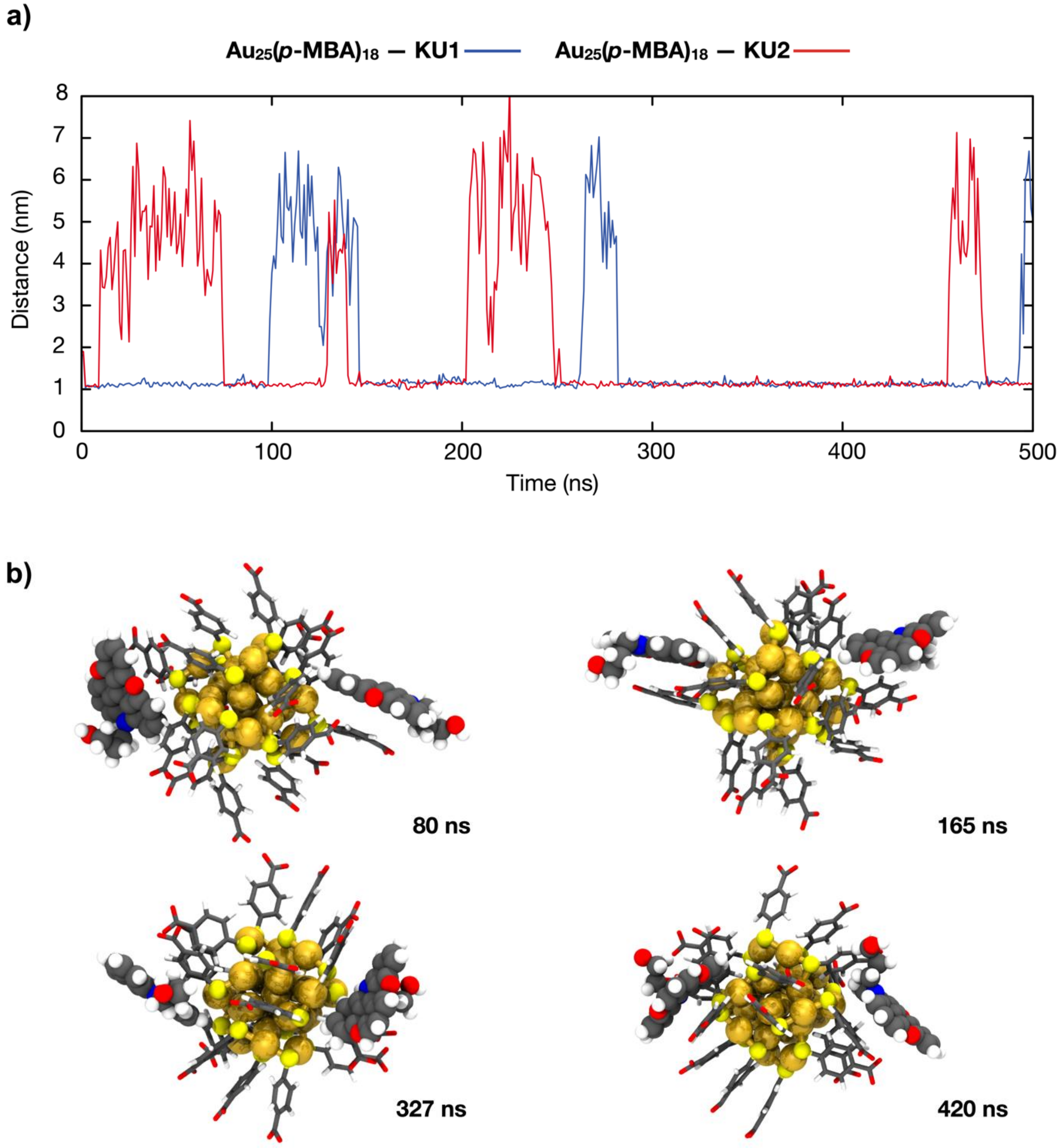

Figure S13. Distance analysis of the Au_{25}(*p*-MBA)$_{18}$+KU complex as a function of simulated time when KU dyes are placed at 1.8 nm with respect to the center of mass of Au_{25}(*p*-MBA)$_{18}$ cluster. (a) distance between the Au_{25}(*p*-MBA)$_{18}$ cluster and each KU dye monitored over the entire 500-ns MD trajectory. (b) representative snapshots extracted from the MD trajectory (time intervals: 75-98 ns, 150-200 ns, 290-450 ns) when both KU dyes interact with the *p*-MBA ligands as a monomer and remain equidistant from the nanocluster.

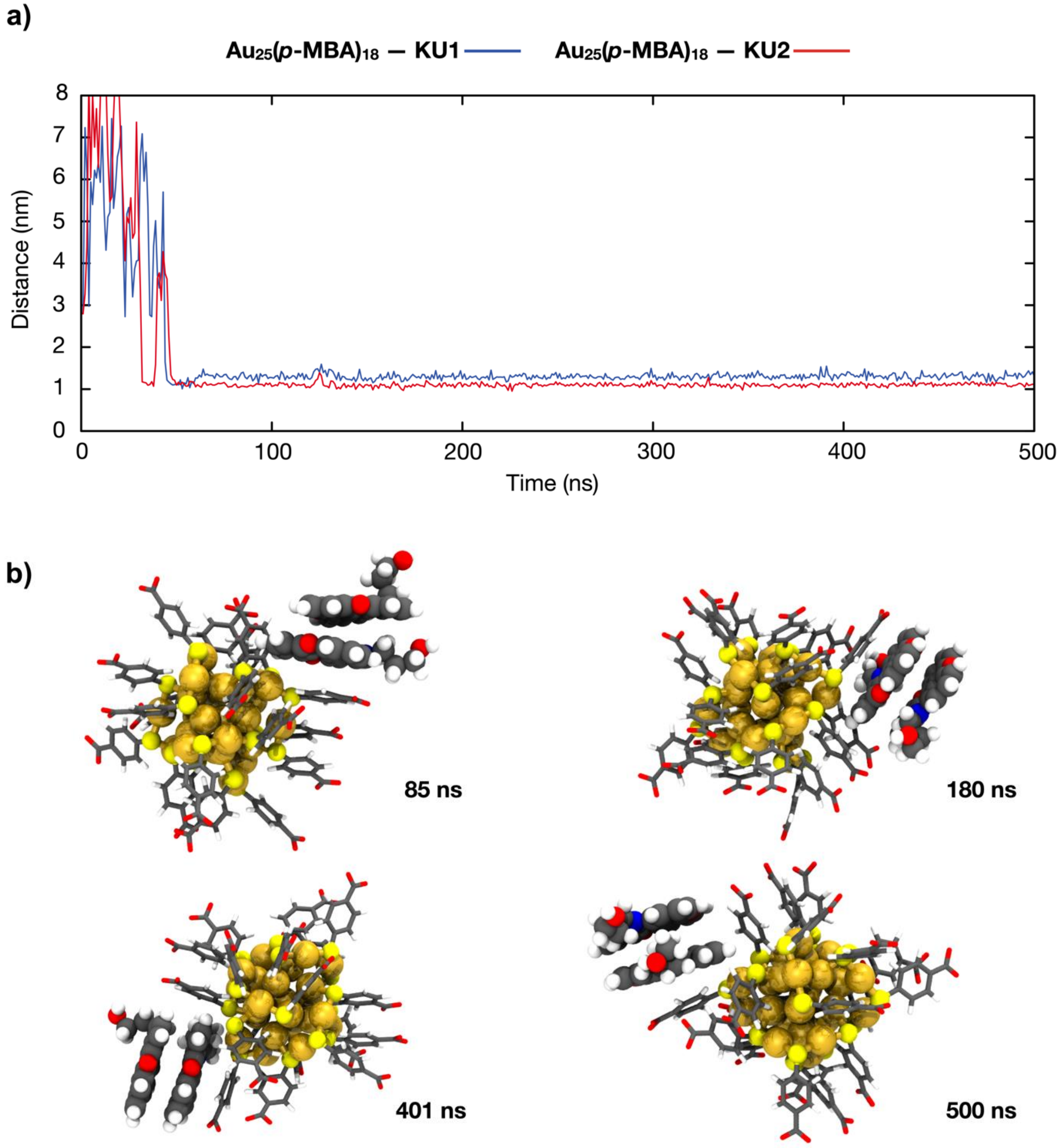

Figure S14. Distance analysis of the Au_{25}(p-MBA)$_{18}$+KU complex as a function of simulated time when KU dyes are placed at 2.8 nm with respect to the center of mass of Au_{25}(p-MBA)$_{18}$ cluster. (a) distance between the Au_{25}(p-MBA)$_{18}$ cluster and each KU dye monitored over the entire 500-ns MD trajectory. (b) representative snapshots extracted from the MD trajectory (time interval: 60-500 ns) when the KU dyes interact with the nanocluster as a π-stacked dimer.

References

1) Pyo, K.; Matus, M. F.; Malola, S.; Hulkko, E.; Alaranta, J.; Lahtinen, T.; Häkkinen, H.; Pettersson, M. Tailoring the interaction between a gold nanocluster and a fluorescent dye by cluster size: creating a toolbox of range-adjustable pH sensors. *Nanoscale Adv*. **2022**, *4*, 4579-4588.
2) Zhao, L.; Pérez Lustres, J. L.; Farztdinov, V.; Ernsting, N. P. Femtosecond fluorescence spectroscopy by upconversion with tilted gate pulses. *Phys. Chem. Chem. Phys.* **2005**, *7*, 1716-1725.
3) Zhang, X.-X.; Würth, C.; Zhao, L.; Resch-Genger, U.; Ernsting, N. P.; Sajadi, M. Femtosecond broadband fluorescence upconversion spectroscopy: Improved setup and photometric correction. *Rev. Sci. Instrum.* **2011**, *82*, 063108.
4) Gerecke, M.; Bierhance, G.; Gutmann, M.; Ernsting, N. P.; Rosspeintner, A. Femtosecond broadband fluorescence upconversion spectroscopy: Spectral coverage versus efficiency. *Rev. Sci. Instrum.* **2016**, 87, 053115.
5) Piel, J.; Beutter, M.; Riedle, E. 20–50-fs pulses tunable across the near infrared from a blue-pumped noncollinear parametric amplifier. *Opt. Lett.* **2000**, *25*, 180-182.
6) Hamm, P.; Kaindl, R. A.; Stenger, J. Noise suppression in femtosecond mid-infrared light sources. *Opt. Lett.* **2000**, *25*, 1798-1800.
7) Abraham, M. J.; Murtola, T.; Schulz, R.; Páll, S.; Smith, J. C.; Hess, B.; Lindahl, E. GROMACS: High performance molecular simulations through multi-level parallelism from laptops to supercomputers. *SoftwareX* **2015**, *1*, 19-25.
8) Pohjolainen, E.; Chen, X.; Malola, S.; Groenhof, G.; Hakkinen, H. A unified AMBER-compatible molecular mechanics force field for thiolate-protected gold nanoclusters. *J. Chem. Theory Comput.* **2016**, *12*, 1342-1350.
9) Berendsen, H. J.; Postma, J. V.; Van Gunsteren, W. F.; DiNola, A. R. H. J.; Haak, J. R. Molecular dynamics with coupling to an external bath. *J. Chem. Phys.* **1984**, *81*, 3684-3690.
10) Bussi, G.; Parrinello, M. Stochastic thermostats: comparison of local and global schemes. *Comput. Phys. Commun.* **2008**, *179*, 26-29.
11) Parrinello, M.; Rahman, A. Polymorphic transitions in single crystals: A new molecular dynamics method. *J. Appl. Phys.* **1981**, *52*, 7182-7190.
12) Darden, T.; York, D.; Pedersen, L. Particle mesh Ewald: An $N \cdot \log(N)$ method for Ewald sums in large systems. *J. Chem. Phys.* **1993**, *98*, 10089-10092.
13) Hess, B.; Bekker, H.; Berendsen, H. J.; Fraaije, J. G. LINCS: A linear constraint solver for molecular simulations. *J. Comput. Chem.* **1997**, *18*, 1463-1472.
14) Humphrey, W.; Dalke, A.; Schulten, K. VMD: Visual molecular dynamics. *J. Mol. Graph. Model.* **1996**, *14*, 33-38.
15) Würth, C.; Grabolle, M.; Pauli, J.; Spieles, M.; Resch-Genger, U. Relative and absolute determination of fluorescence quantum yields of transparent samples. *Nat. Protoc.* **2013**, *8*, 1535-1550.
16) García, B.; Ibeas, S.; Ruiz, R.; Leal, J. M.; Biver, T.; Boggioni, A.; Secco, F.; Venturini, M. Solvent effects on the thermodynamics and kinetics of coralyne self-aggregation. *J. Phys. Chem. B* **2009**, *113*, 188-196.
17) Padmapriya, K.; Barthwal, R. Nuclear magnetic resonance based structure of the protoberberine alkaloid coralyne and its self-association by spectroscopy techniques. *J. Pharm. Anal.* **2019**, *9*, 437-448.

18) Chaires, J. B.; Dattagupta, N.; Crothers, D. M. Self-association of daunomycin. *Biochemistry* **1982**, *21*, 3927-3932.
19) Agrawal, P.; Barthwal, S. K.; Barthwal, R. Studies on self-aggregation of anthracycline drugs by restrained molecular dynamics approach using nuclear magnetic resonance spectroscopy supported by absorption, fluorescence, diffusion ordered spectroscopy and mass spectrometry. *Eur. J. Med. Chem.* **2009**, *44*, 1437-1451.
20) Shao, C.; Grüne, M.; Stolte, M.; Würthner, F. Perylene bisimide dimer aggregates: Fundamental insights into self-assembly by NMR and UV/Vis spectroscopy. *Chem. Eur. J.* **2012**, *18*, 13665-13677.
